# Tumor microenvironment-responsive fenton nanocatalysts for intensified anticancer treatment

**DOI:** 10.1186/s12951-022-01278-z

**Published:** 2022-02-05

**Authors:** Yandong Wang, Fucheng Gao, Xiaofeng Li, Guiming Niu, Yufei Yang, Hui Li, Yanyan Jiang

**Affiliations:** 1 Key Laboratory for Liquid-Solid Structural Evolution & Processing of Materials (Ministry of Education), School of Materials Science and Engineering, Shandong University, Jinan, Shandong 250061 People’s Republic of China; 2 Shenzhen Research Institute of Shandong University, Shenzhen, Guangdong 518057 People’s Republic of China

**Keywords:** Nanocatalyst, Fenton reaction, Tumor microenvironment, Multi-mode therapy, Cancer treatment

## Abstract

Chemodynamic therapy (CDT) based on Fenton or Fenton-like reactions is an emerging cancer treatment that can both effectively fight cancer and reduce side effects on normal cells and tissues, and it has made important progress in cancer treatment. The catalytic efficiency of Fenton nanocatalysts(F-NCs) directly determines the anticancer effect of CDT. To learn more about this new type of therapy, this review summarizes the recent development of F-NCs that are responsive to tumor microenvironment (TME), and detailedly introduces their material design and action mechanism. Based on the deficiencies of them, some effective strategies to significantly improve the anticancer efficacy of F-NCs are highlighted, which mainly includes increasing the temperature and hydrogen peroxide concentration, reducing the pH, glutathione (GSH) content, and the dependence of F-NCs on acidic environment in the TME. It also discusses the differences between the effect of multi-mode therapy with external energy (light and ultrasound) and the single-mode therapy of CDT. Finally, the challenges encountered in the treatment process, the future development direction of F-NCs, and some suggestions are analyzed to promote CDT to enter the clinical stage in the near future.

**Graphical Abstract:**

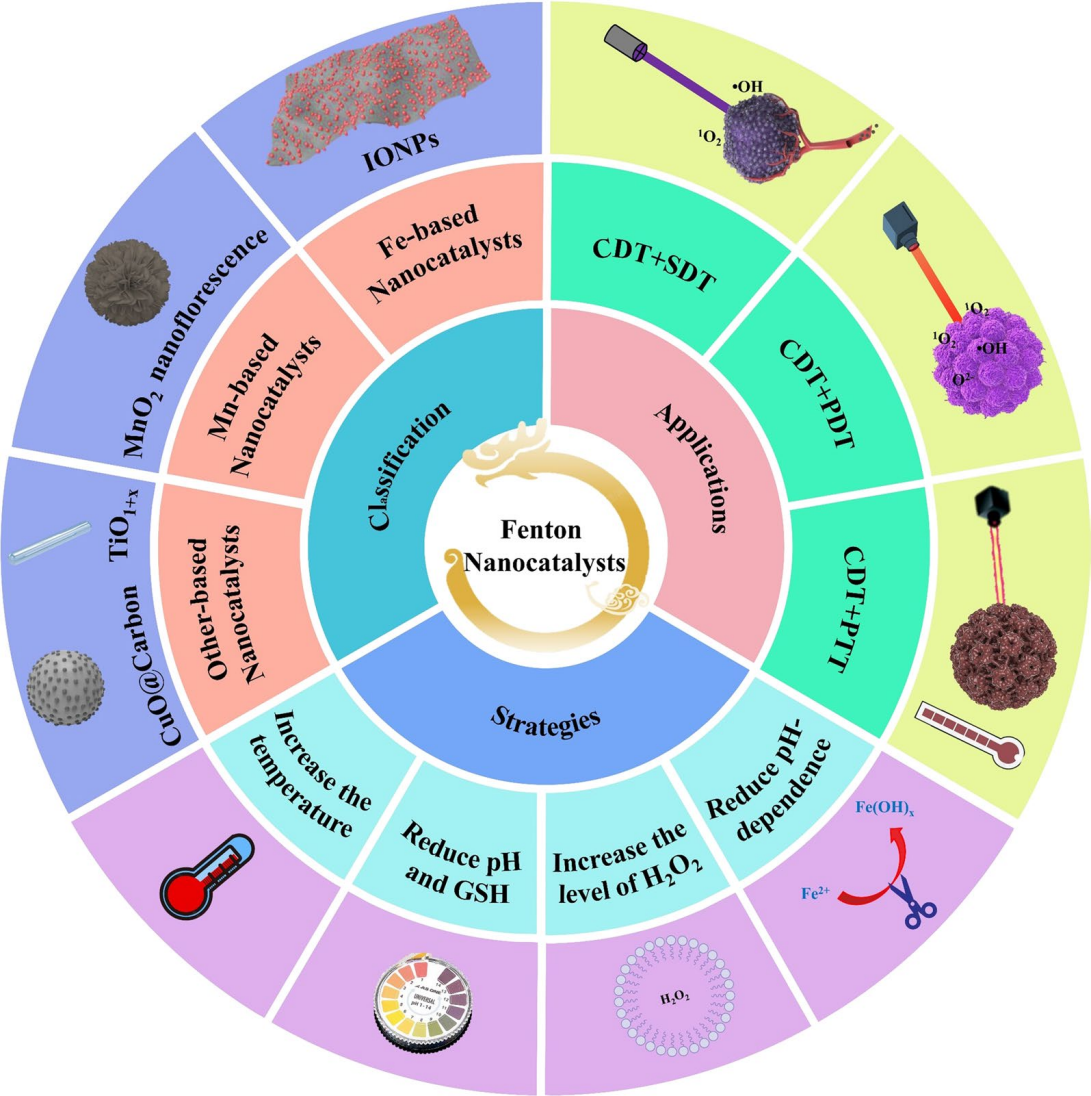

**Supplementary Information:**

The online version contains supplementary material available at 10.1186/s12951-022-01278-z.

## Introduction

Malignant tumor is one of the main causes of death in the world. It has become a major disease that seriously endangers human life and health and restricts social and economic development [1, 2]. Traditional methods of cancer treatment mainly include surgical resection, radiotherapy, and chemotherapy [3, 4]. However, conventional treatments have many limitations (such as low selectivity, easy recurrence, large side effects, and so on) [5]. Fortunately, nanotechnology shows great potentials to improve the anticancer effect and reduce the side effects, and various nanomedicines are widely applied to different new therapeutic methods, including hyperthermia therapy, sonodynamic therapy (SDT), immunotherapy, and chemodyanic therapy (CDT) [6]. Among them, CDT has attracted much attention in recent years due to its strong oxidative lethality to cells and specific suborganelles [7].

CDT is an emerging and minimally invasive cancer treatment, it is defined as the transformation of endogenous H_2_O_2_ through Fenton or Fenton-like reactions into highly harmful hydroxyl radical (•OH), which is known as the most oxidizing reactive oxygen species (ROS), and can induce massive apoptosis of tumor cells by damaging DNA and inactivating proteins [8]. Compared with normal cells, cancer cells have a unique way of proliferation, metabolic activity, and mitochondrial dysfunction so that the tumor tissue has a unique structure and physical properties. Especially, the content of hydrogen peroxide (H_2_O_2_) in tumor tissues is far higher than that of normal tissues [9]. CDT relies on the higher expression of H_2_O_2_ in tumors, so this method is highly selective and can reduce the damage to normal tissues [10, 11]. However, the low efficiency of CDT limits its potential clinical applications.

**Fig. 1:**
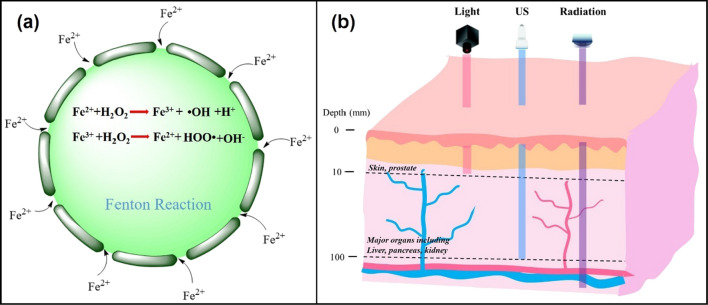
**a** Schematic diagram of the Fenton reaction equation. **b** Diagram of penetration depth of light, ultrasound (US) and radiation to human tissues. The light penetrates to a depth of 10 mm, which just only be used for the treatment of shallow tumors, while ultrasound can be used for the treatment of deep tumors with a tissue penetration depth of 10 cm, which can reach to major organs in the body. Although the penetration depth of radiation is deeper than the light and US, it can cause damage to the normal tissue

Fenton and Fenton-like reactions are the basis of CDT, which determine the efficiency of this treatment, the equation of Fenton reaction is shown in Fig. 1a [8]**.** The discovery of Fenton reaction comes from the British scientist H. J. H. Fenton. In 1983, he first proved that H_2_O_2_ in acidic environment has the ability to oxidize various organic substances under the catalysis of iron ions, and this technology has widely applied to the field of wastewater treatment [12]. Inspired by this technology, various metals with Fenton-like effect have been developed and applied to cancer treatment, such as Au [13], Ag [14], Cu [15], Mn [16], and so on. However, the tumor is not the best place for Fenton reaction, which greatly reduces the efficiency of CDT. To improve the therapeutic effect of chemical kinetics, three conditions must be met to produce sufficient hydroxyl radicals (•OH). First, sufficient hydrogen peroxide concentration. The concentration of H_2_O_2_ in the tumor microenvironment (TME) is not enough to continuously produce •OH [17]. Therefore, increasing the level of H_2_O_2_ in the TME is the main method to solve this problem. Second, the generation rate of •OH must be fast enough to produce strong oxidation to the tumor in a short time, so as to avoid the resurrection of cancer cells. The generation rate of •OH can be adjusted by changing the reaction conditions (such as temperature and pH) and optimizing the structure and composition of F-NCs [18, 19]. Third, •OH produced by Fenton or Fenton-like reactions should attack cancer cells directly as much as possible, rather than being captured by reducing substances in the TME, such as (GSH) [20].

In addition to the above strategies, another direct way to improve the therapeutic effect of CDT is multi-mode therapy. For example, CDT combined with photothermal therapy (PTT), photodynamic therapy (PDT), or SDT. The combination of CDT and PTT can be realized by using F-NCs with photothermal conversion ability. In the combined treatment of CDT/PTT, the temperature of TME can be increased to accelerate the catalytic effect of Fenton reagents and finally enhance the therapeutic effect of CDT. The combination of CDT/PDT is mainly realized by loading photosensitizer on Fenton reagent, or the transition metal ions with Fenton or Fenton-like effect are coordinated and self-assembled with photosensitizers which can increase the concentration of ROS in TME under the excitation of light [21, 22]. PDT and PTT both use light as external energy to enhance the therapeutic effect. However, the fatal disadvantage of light in the treatment process is the low maximum penetration depth in the body (about 10 mm), which greatly restricts the application of PDT and PTT [23]. In order to overcome the defect of insufficient penetration ability of light in the body, researchers use ultrasound (US) to replace light and the maximum penetration depth of ultrasound is about 10 cm (Fig. 1b) [20]. It is possible to combine SDT and CDT to treat deep tumors in vivo, which solves the defect of CDT that cannot produce ROS continuously.

In recent years, the new applications of F-NCs in cancer treatment make the Fenton reaction, an ancient reaction, flourish again. However, CDT is still in the preliminary stage with some deficiencies to overcome. This paper elaborates the preparation process and mechanism of F-NCs in detail and summarizes the recent development of F-NCs applied to cancer treatment. According to the shortcomings of F-NCs, some strategies that can improve the anticancer effect of them have been proposed. Especially, the applications of F-NCs in other therapeutic methods are summarized and the development directions of them in the future are prospected. This review aims to improve researchers’ understanding of F-NCs (such as reaction conditions, properties, and mechanisms). More importantly, it provides some important strategies for improving their therapeutic efficiency.

## Classification and featured chemistry of F-NCs

CDT, based on the weak acid of TME as reaction condition, uses H_2_O_2_ as raw material and transition metal nanomaterials as the catalyst to initiate Fenton or Fenton-like reactions in cancer cells so as to catalyze H_2_O_2_ to produce •OH, O_2_^2−^, ^1^O_2_ and other substances with strong oxidation to induce cell apoptosis. The essence of the Fenton reaction is the chain reaction between Fe^2+^ and H_2_O_2_, which can promote the generation of ROS [24–26]. In addition, Mn^2+^, Ti^3+^, and Cu^+^ can also catalyze the decomposition of H_2_O_2_ to produce ROS, which is called Fenton-like reaction [27]. Catalysts play a crucial role in Fenton or Fenton-like reactions, so the design of catalysts is very important. The preparation of different kinds of F-NCs will be described in detail in the following subsections.

### Fe-based F-NCs

Iron element is widely present in various tissues and organs of the human body, which plays a significant role in oxygen transport, glucose metabolism, and ATP generation, and shows superior biocompatibility. Therefore, iron-based materials have been widely used in the biological field and show high biosafety [28]. Moreover, iron-containing F-NCs have special magnetic properties and are effective contrast agents for magnetic resonance imaging (MRI), which can enhance the detection of tumor lesions in vivo [29]. Based on these advantages, Fe-based F-NCs have been extensively studied in CDT.

**Fig. 2:**
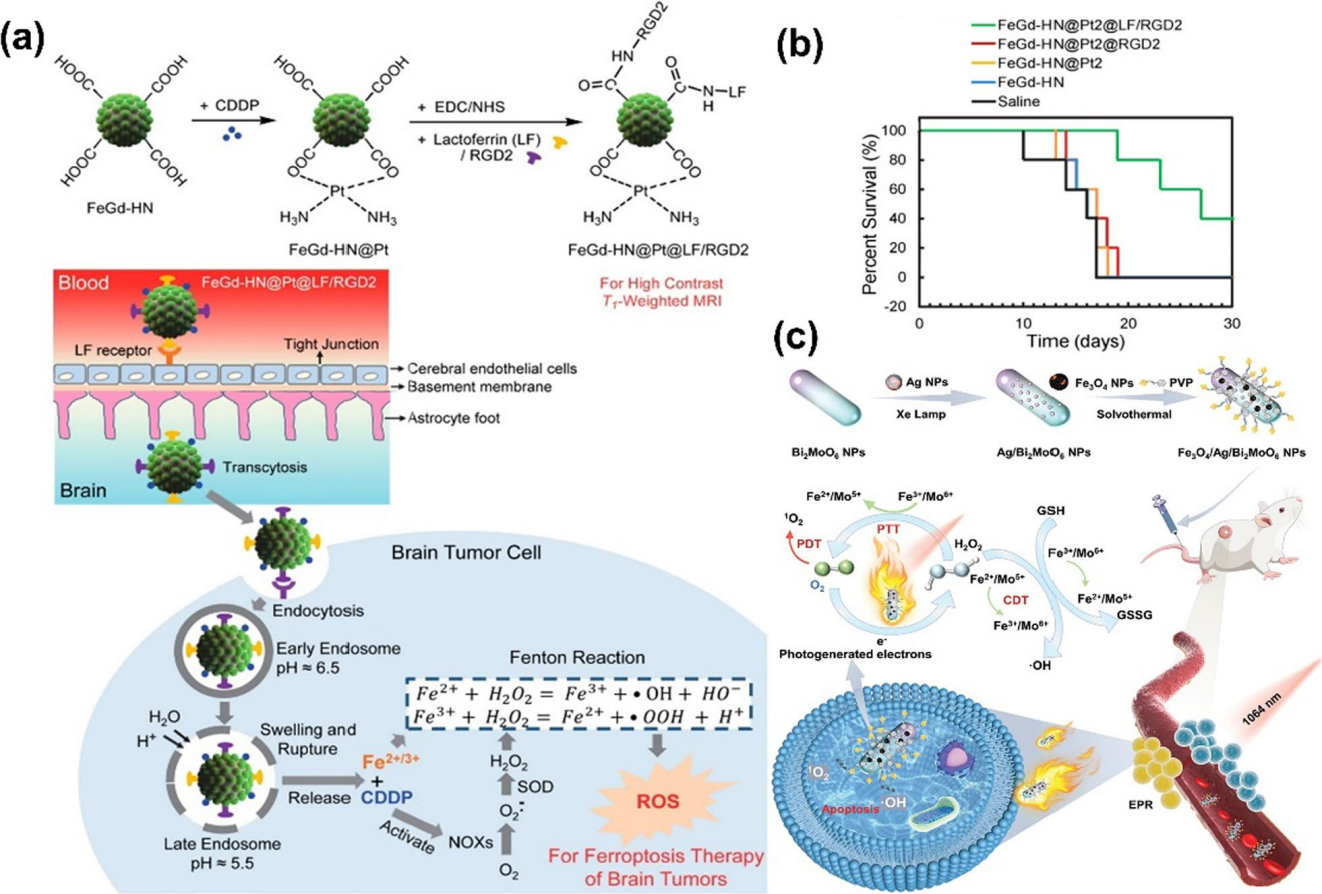
**a** The synthesis and cell endocytosis mechanism schematic diagram of FeGd-HN@Pt@LF/RGD_2_ NPs and intracellular effects of FeGd-HN@Pt@LF/RGD_2_ NPs on cancer cells [31]. **b** Percent survival of the mice bearing orthotopic brain tumors after treatment. Treatment with FeGd-HN@Pt_2_@LF/RGD_2_ significantly extended the survival of tumor-bearing mice. Reproduced with permission. [31] Copyright 2018, American Chemical Society. **c** Schematic illustration for the fabrication of FAB NPs and the mechanisms of synergistic CDT/PDT/PTT therapy. Reproduced with permission. [33] Copyright 2021 Wiley‐VCH

Iron oxide nanomaterials are an important part of Fe-based F-NCs, which are widely used in CDT [30]. More importantly, they also play a positive role in regulating TME and tumor metabolism and promoting tumor therapy. A recent Fe-based Fenton nanocatalyst involving Fe_3_O_4_ nanoparticles (FeGd-HN@Pt@LF/RGD_2_ NPs) showed significant anti-tumor effects [31]. In this system, the cis-platinum (CDDP) was loaded on Fe_3_O_4_/Gd_2_O_3_ hybrid NPs, and modified the hybrid NPs with lactoferrin (LF) and RGD dimer (RGD_2_), as shown in Fig. 2a. The LF on the surface of these NPs could help FeGd-HN@Pt@LF/RGD_2_ nanoplatform cross the blood–brain barrier and then this nanoplatform could be specifically internalized by cancer cells upon integrin αvβ3 binding. The released Fe^2+^ and Fe^3+^ directly participated in the Fenton reaction, while CDDP indirectly produced H_2_O_2_ in cancer cells, strengthening the Fenton response in cancer treatment. The specific therapeutic mechanism of the FeGd-HN@Pt@LF/RGD_2_ nanoplatform is shown in Fig. 2a. According to the in vivo experiment, Fig. 2b showed that FeGd-HN@Pt_2_@LF/RGD_2_ NPs can significantly extend the survival of tumor-bearing mice. In addition, ultrafine Fe_3_O_4_ NPs are also known as superparamagnetic iron oxide NPs (IONPs), which are good MRI T2 contrast agents for intravenous administration. It has unique advantages for cell labeling in vivo tracer experiments [32]. For example, an “all-in-one” Fe_3_O_4_/Ag/Bi_2_MoO_6_ (FAB) nano platform can be used as an MRI contrast agent, which is helpful to observe the pharmacokinetic characteristics of FAB NPs and determine the optimal treatment time [33]. In brief, FAB NPs were synthesized by three steps, including hydrothermal synthesis of Bi_2_MoO_6_ NPs, photoreduction of Ag NPs, and solvent doping of Fe_3_O_4_ together with a surface covering of hydrophilic polyvinylpyrrolidone (PVP), as shown in Fig. 2c. In this work, the incorporation of Fe_3_O_4_ and Ag could enhance the photocatalytic activity, ferromagnetic and photothermal effect of UV-adsorbing Bi_2_MoO_6_ NPs. Moreover, Fe_3_O_4_ endowed FAB NPs with the Fenton effect and the ability of MRI. Finally, FAB NPs could highly inhibit tumor growth, which was attributed to the synergy between CDT/PTT/PDT, as well as the sustainable and self-complementary anti-tumor strategy caused by the coupling effect between cascaded nano catalytic reaction and multi-enzyme activity, as shown in Fig. 2c.

**Fig. 3:**
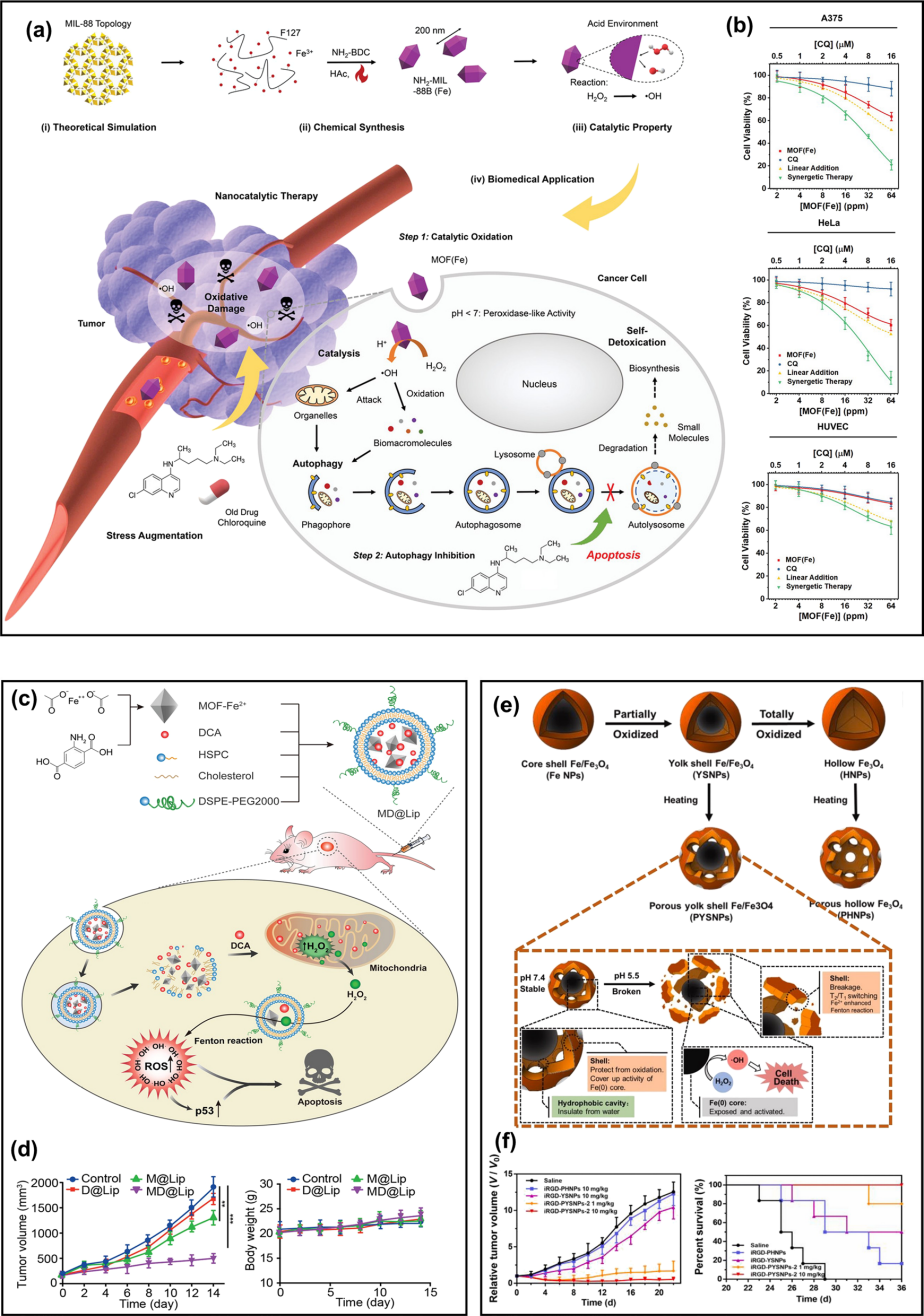
**a** The preparation and action mechanism of NH_2_-MIL-88b (Fe) in cancer cells [35]. **b** The apoptosis of cancer cells (A375 and HeLa) and normal cells (HUVEC) induced by synergistic therapy. Reproduced with permission. [35] Copyright 2020, WILEY–VCH. **c** The synthesis process of MD@Lip and the action mechanism of MD@Lip in cancer cells [38]. **d** The tumor volume and body weights of mice during 14-day treatment. Reproduced with permission. [38] Copyright 2019, WILEY–VCH. **e** The preparation process of PYSNPs and PHNPs and the mechanism of pH-activated PYSNPs releasing Fe [41]. **f** The relative tumor volume and survival curves of mice after different treatments. Reproduced with permission. [41] Copyright 2019, WILEY–VCH

In addition to iron oxide nanomaterials, some iron-containing metal–organic frameworks (MOFs) nanocatalysts also have a good Fenton effect and have been applied in cancer treatment. Compared with iron oxides, iron-containing MOF has better flexibility, responsiveness, and dispersion. Moreover, MOF has a better ability to penetrate cell membranes, which can enhance the treatment effect or imaging capability on the basis of enhancing permeability and retention effect (EPR) [34]. Recently, an iron-containing MOF(Fe) nanosystem (NH_2_-MIL-88B(Fe)) with catalase activity was fabricated to inhibit autophagy and enhance ROS-induced oxidative damage [35]. The structure and catalytic mechanism of NH_2_-MIL-88B (Fe) are shown in Fig. 3a. These NCs could promote the generation of highly oxidized •OH in cancer cells under an acid environment, among which chloroquine is a classical autophagy inhibitor. As can be seen in Fig. 3b, the results of the combined effect of chloroquine and NCs on cancer cells and normal human cells verified that chloroquine and NCs could synergistically enhance the anticancer effect compared with chloroquine and NCs alone. Because the synergetic therapy could effectively block autophagy so that cancer cells cannot extract their own components to detoxify and enhance their own metabolism, and eventually die from the strong oxidation of ROS under the catalysis of NCs. In human umbilical vein endothelial cells (HUVECs), the negligible effect of single MOF(Fe) therapy or synergistic therapy indicated that MOF(Fe) had high therapeutic specificity due to the absence of H_2_O_2_ in normal cells. MOF-Fe composites have different morphology and composition, which consequently results in higher catalytic activity [36]. However, MOF-Fe nanomaterials also have some disadvantages. For example, the activation of their catalytic activity has high requirements on the existing environmental conditions. The pH in the TME is 6.5–7, while the catalytic activity of MOF-Fe is extremely low in this physiological environment, which will limit the scope of its biological applications [37]. Some studies have shown that MOF-Fe catalysis of Fenton reaction can only be carried out in acidic media (pH is 2.0–5.0) [38]. To solve this problem, researchers designed pH-responsive MOFs that can regulate the TME. For example, an iron-containing MOF(Fe^2+^) nanosystem that contained dichloroacetic acid (DCA) can break the limitation of pH [38] (Fig. 3c). DCA is an analog of acetic acid, which can not only reduce the mitochondrial membrane potential but also participate in the oxidation of glucose and increase the concentration of H_2_O_2_ in the tumor. Moreover, DCA is a strong organic acid with a pKa value of 1.35. The addition of DCA could regulate the pH in the TME, activate the maximum catalytic activity of MOF-Fe^2+^ NPs on the Fenton reaction in the tumor, and decompose H_2_O_2_ in the tumor to produce more toxic ROS. Liposomes were coated on the surface of MOF-Fe^2+^ NPs, which can increase their solubility. Figure 3d indicated that MOF-Fe^2+^-DCA@Liposomes NCs (MD@Lip NCs) have low biological toxicity. More importantly, the combination of DCA and MOF-Fe^2+^ could highly improve anticancer efficacy.

The introduction of organic acids to regulate TME is an innovative idea, but the precise control of the number of organic acids is a major problem in the research. Therefore, in addition to adding acidic substances that can regulate the TME to the NCs, the catalytic activity of F-NCs in the TME can also be increased by improving their properties. Recent studies have shown that zero-valent iron (Fe(0)) is a more active F-NC and has been used for wastewater decontamination [39, 40]. However, due to the unstable chemical properties of nanoscale Fe(0), which is easy to be oxidized, it is necessary to construct a stable nanoplatform that can effectively transport Fe(0). Liang et al. [41] fabricated a Fe/Fe_3_O_4_ nanoplatform with a porous yolk-shell structure as shown in Fig. 3e. The porous Fe_3_O_4_ shell could protect the Fe(0) nucleus (5 nm) from oxidation in the normal physiological environment for several weeks. In the acidic TME, the pores were etched and ruptured, and Fe(0) was released. Through the study of Fe-release about different Fe_3_O_4_ shells under different pH conditions, porous hollow Fe_3_O_4_ NPs (PHNPs) was stable under both neutral and acidic conditions, so Fe(0) was a major source of Fe-release from the nano platform during treatment in the condition of weak acidity. Compared with other NPs, porous yolk-shell Fe/Fe_3_O_4_ NPs (PYSNPs) could effectively inhibit tumor growth and significantly improve the survival time of mice, which depended on the high catalytic activity of Fe (0), as shown in Fig. 3f.

In addition to the iron-based nanomaterials described above, other iron-containing nanomaterials have also been used for cancer treatment, such as natural biomineral ferrihydrite [42], ferric hydrogels [43], iron sulfides [44], and organometallic compounds ferrocene [45]. Here, the representative F-NCs in recent years are summarized, as shown in (Additional file 1: Table S1).

### Mn-based F-NCs

Compared with iron oxides, manganese oxides have a stronger oxidation capacity, so manganese oxide NCs have more advantages in consuming GSH in tumor cells. Similar to Fe^2+^, Mn^2+^ is also an effective contrast agent for T1-weighted magnetic resonance imaging (T1-MRI) for tumor detection, allowing real-time detection of the distribution of nano-catalysts in vivo [46, 47]. Mn is an essential trace element for the human body, and Mn^2+^ is a water-soluble ion that can be rapidly excreted through the kidneys. Based on these advantages, Mn-based nanomaterials are also used as NCs with a Fenton-like effect in cancer treatment.

**Fig. 4:**
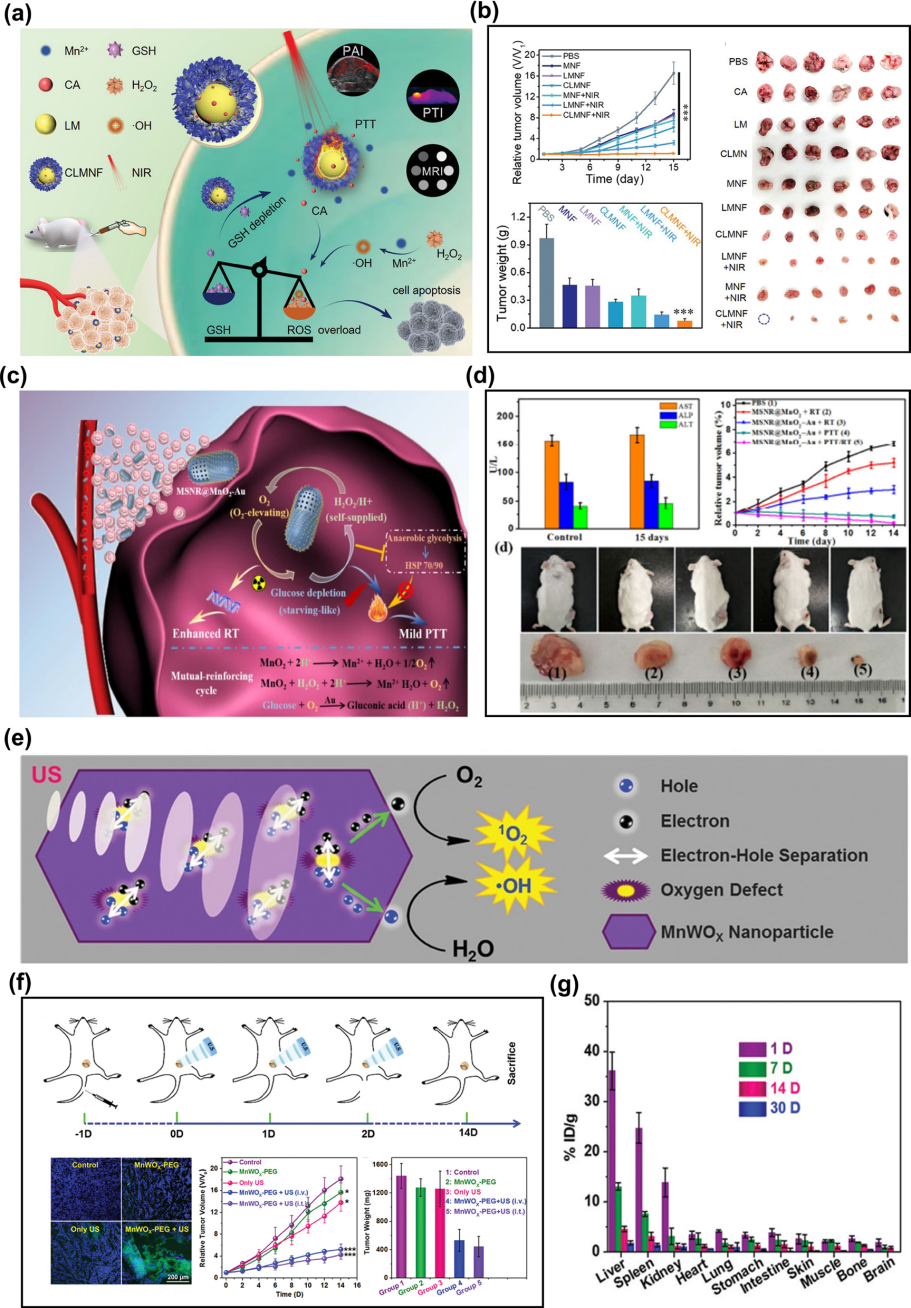
**a** Diagram of the composition and therapeutic mechanism of CLMNF nanoparticles [48]. **b** The relative tumor volume, tumor weight, and photographs of tumor tissues of CT26 tumor-bearing mice with various treatments (n = 6, mean ± SD ***p < 0.001). Reproduced with permission. [48] Copyright 2020, Wiley–VCH. **c** The therapeutic mechanism of MnO_2_-Au@SiO_2_ nanoplatforms in solid tumors [49]. **d** In vivo toxicology assessment of MnO_2_-Au@SiO_2_ NPs; the relative tumor volume and photographs of tumor-bearing mice and tumor tissues from tumor-bearing mice after different treatment. Reproduced with permission. [49] Copyright 2020, Tsinghua University Press and Springer-Verlag GmbH Germany, part of Springer Nature. **e** The mechanism of MnWO_x_ leads to increase ROS production [50]. **f** Schematic of the in vivo SDT procedure on mice and fluorescence images of DCFH-DA-stained tumor slices collected from mice 24 h post-treatment and tumor growth curves and average weights of tumors after various treatments [50]. **g** Distribution of W levels in mice at different times based on inductively coupled plasma measurement. Reproduced with permission. [50] Copyright 2019, Wiley–VCH

MnO_2_ is an important component of Mn-based NCs. MnO_2_ can undergo a redox reaction with GSH in the body to reduce the level of GSH in cancer cells and produce Mn^2+^. Mn^2+^ can catalyze the decomposition of H_2_O_2_ to produce ROS and induce apoptosis of cancer cells. For example, Liu et al. [48] used liquid metal (Lm: 75%Ga and 25%In) NPs as templates to design a yolk-shell structure of Lm@MnO_2_ (LMN). LMNs were then loaded with cinnamaldehyde (CA) to form CLMN and further coated with hyaluronic acid (HA) to construct CA&LM@MnO_2_-HA nanoflowers (CLMNF) for cancer targeted therapy. CLMNF particles rapidly consumed GSH and produced Mn^2+^, which further promoted the conversion of H_2_O_2_ to ·OH to intensify the death of cancer cells. Besides, LM endured the NPs with good near-infrared photothermal conversion ability. The composition and therapeutic mechanism of CLMNF NPs in vivo are shown in Fig. 4a. Figure 4b proved that the combination of CLMNF and NIR could make cancer cells apoptosis efficiently, and the tumor in mice could be basically eliminated, due to the combined action of PTT/CDT. Yang et al. [49] also verified that the combination of MnO_2_ and other metals has the function of multi-mode therapy. MnO_2_ and ultra-small gold NPs were deposited on mesoporous silica nanorods, and subsequently, a MnO_2_-Au@SiO_2_ nano-reaction platform with multi-modal imaging synergism to improve O_2_ content and heat sensitivity in tumors was prepared. MnO_2_ could catalyze the decomposition of H_2_O_2_ to produce ROS, and Au could stably and efficiently oxidize glucose in TME, thus making tumor cells sensitive to thermal ablation, as shown in Fig. 4c. Comparing the liver and kidney function indicators of mice injected or not injected with MnO_2_-Au@SiO_2_, it is indicated that this nanoplatform had a good hepatic and kidney safety profile. More importantly, this nanoplatform could effectively promote cancer cell apoptosis, as can be seen in Fig. 4d. It is suggested that the combination of MnO_2_ nanomaterials and other metals to construct a multifunctional nano platform to induce oxidative/heat stress damage in cancer cells is expected to be an effective anti-cancer treatment strategy.

In addition to MnO_2_ NCs, bimetallic manganese oxides with anoxic structures have excellent therapeutic effects. The hypoxic structure can be used as an electronic trap to prevent electron–hole recombination, improve the quantum yield of ROS, and release Mn^2+^ that can catalyze the decomposition of H_2_O_2_ in solid tumors to improve the therapeutic effect. Constructing anoxic structures to improve the production of ROS is a method that has been less studied at present, and it is highly innovative. An ultra-small bimetallic oxide MnWO_x_ nano platform prepared by Gong’s group displayed a strong ability of ROS generation due to the hypoxic structures in MnWO_x_, which provided electron capture sites to prevent electron–hole recombination (Fig. 4e) [50]. Moreover, MnWO_x_ could consume GSH in tumors, release Mn^2+^ in an acidic environment and further increase the level of ROS, finally realizing the combination of CDT and SDT. According to the in vivo study, the synergy of CDT and SDT can highly improve the antitumor ability (Fig. 4f). In this nanoplatform, the Mn and W elements enable MnWO_x_ NPs to display considerable contrast in magnetic resonance and computed tomography imaging, which could be used to track the accumulation of NPs in animals. Moreover, the size of MnWO_x_ NPs is very small with an average diameter of 5.74 ± 1.66 nm, which could be rapidly metabolized in mice. The level of W in vivo was measured by inductively coupled plasma emission spectrometry. After 30 days, the retention rate of W is less than 1.7% ID g^−1^. The results in Fig. 4g indicate that MnWO_x_-PEG is a safe sonosensitizer, which has Fenton catalytic ability and no retention in the mice body.

In addition to manganese oxide and bimetallic manganese oxide NCs, there are some other manganese-related NCs with the Fenton effect. Here, we summarize the recent advances in manganese nano catalytic materials for the treatment of cancer, as shown in (Additional file 1: Table S2).

### Other metal-based F-NCs

**Fig. 5:**
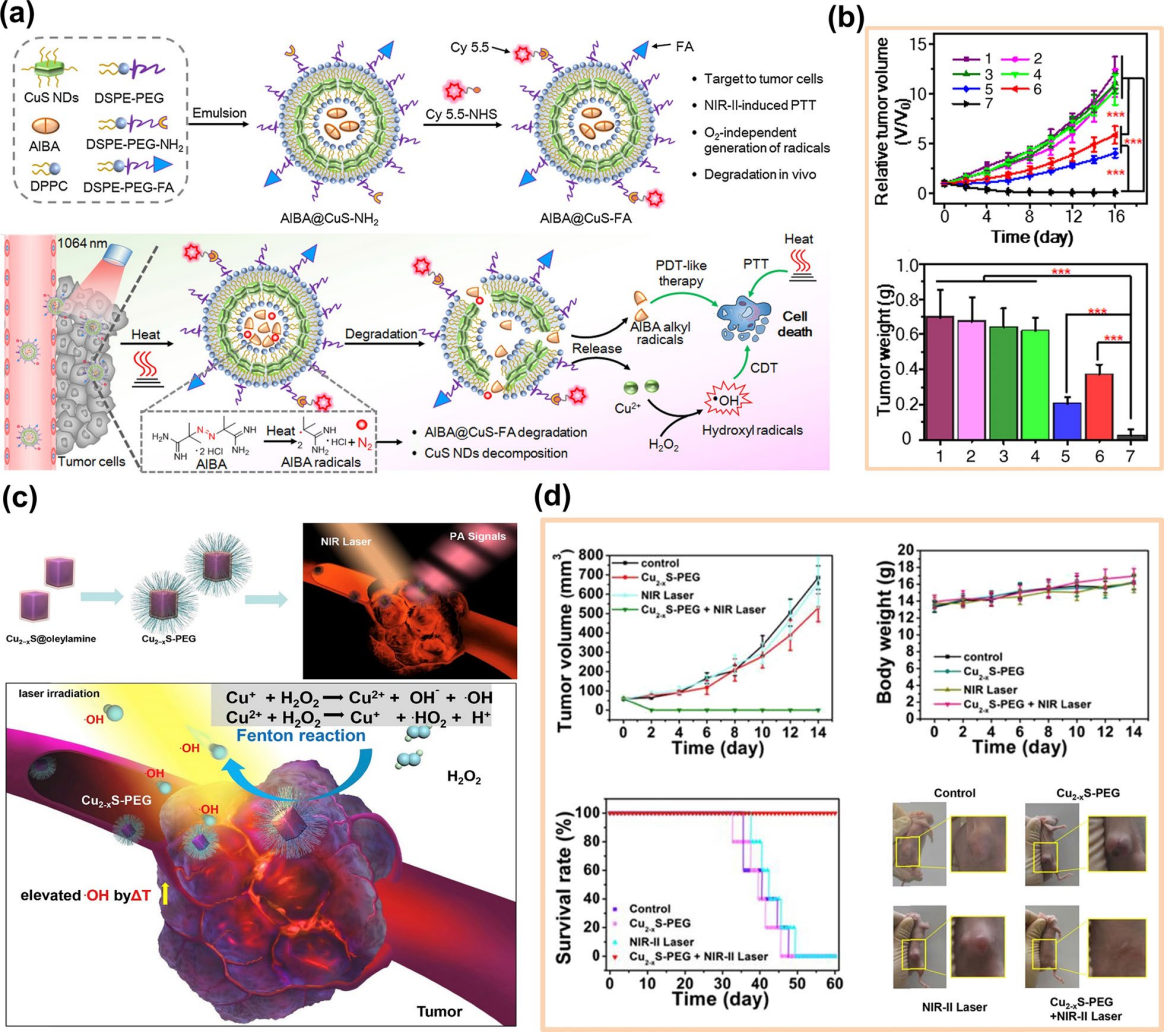
**a** The general design, preparation, degradation, and action mechanism of AIBA@CuS-FA NPs [60]. **b** The relative tumor volume and tumor weight of mice on 16^th^ day after different treatments (1. PBS, 2. CuS-FA, 3. AIBA@CuS-FA, 4. PBS + 1064 nm, 5. CuS-FA + 1064 nm, 6. AIBA@CuS-FA + 808 nm, 7. AIBA@CuS-FA + 1064 nm). Reproduced with permission. [60] Copyright 2020, Chinese Chemical Society. **c** Fenton-like reactions of Cu_2-x_S nanoparticles in solid tumors. [61]. **d** The tumor volume and tumor weight growth of nude mice on 14th day, the survival rates of 4T1 tumor-bearing nude mice within 60 days feeding duration, and the digital images of tumors of each group after receiving varied treatments, including control group, Cu_2-x_S-PEG NDs group, NIR-II laser group, and Cu_2-x_S-PEG NDs + NIR-II laser group. Reproduced with permission. [61] Copyright 2019, Elsevier Ltd

In addition to Fe-and Mn-based Fenton reagents, other metals have also been used to catalyze Fenton-like reactions, such as Ti [51], Cu [52], Ag [53], V [54], Pt [55], Co [56], Ru [57] and Au [58], etc. For Cu-based NCs, the MOF derivatives of Cu [59], sulfides of Cu (such as CuS [60] and Cu_2-x_S [61]), copper oxides (such as CuO [62, 63] and Cu_2_O [64]), and copper bimetallic compounds (such as CuSe) [65], are used as catalysts of Fenton-like reactions. Compared with the traditional Fe-based F-NCs, due to the inherent microenvironment response behavior and high biocompatibility of Fe-based F-NCs, the degradation ability of Fe-based F-NCs in vivo is stronger than that of Cu-based F-NCs, and the retention time in vivo is shorter and easier to be discharged from the body [66]. Although some Cu-based F-NCs have been preliminarily proved to be biocompatible, the high accumulation of Cu may cause potential toxicity problems. Therefore, generally speaking, the biological toxicity of Fe-based F-NCs is lower than that of Cu-based F-NCs, so it was considered that the use of Cu-based F-NCs in biomedicine was relatively limited in early times. However, with the improvement of scientific research and technology, researchers found that transition metal Cu plays an irreplaceable role in the biomedical field, such as Cu can enhance angiogenesis and affect liposome/glucose metabolism [67]. The physical and chemical properties of Cu-based F-NCs can meet the needs of various biomedical applications. For example, Cu-based chalcogenides have strong absorption in the near-infrared window and have a photothermal/photodynamic effect. The photothermal effect can induce tissue expansion, which is conducive to the application of Cu-based F-NCs in photoacoustic imaging (PA) and PTT. In addition, Cu-based F-NCs have a higher Fenton reaction rate and can react in a wide range of pH. The Fenton reaction rate of traditional Fe-based F-NCs is 76 m^−1^ s^−1^ and Cu-based F-NCs is 1 × 10^4^ m^−1^ s^−1^ [68–70]. Based on these advantages of Cu, more and more Cu-based F-NCs have been used in cancer treatment in recent years. However, whether Cu-based F-NCs can enter clinical applications ultimately still depends on their toxicity. Fortunately, many Cu-based F-NCs prepared in recent years have been proved to be low toxic or even non-toxic, with high biocompatibility and biosafety. The key to reducing the biological toxicity of Cu-based F-NCs is to avoid the release of copper ions in Cu-based nanosystems before the materials exert their properties. Amphiphilic liposomes are widely used in material modification and drug loading due to their good biocompatibility and low toxicity, Amphiphilic liposome encapsulation of Cu-based F-NCs can hinder the premature release of Cu. For example, the preparation of AIBA@CuS-FA NPs was obtained by encapsulating hydrophilic azo initiator (AIBA) and CuS with amphiphilic liposomes [60]. In this nanosystem, CuS is a nanomaterial with photothermal conversion ability and triggers the thermal decomposition of AIBA into cytotoxic free alkyl groups under laser irradiation. Subsequently, free alkyl could promote the degradation of AIBA@CuS-FA NPs and produce Cu^2+^, which could catalytically decompose H_2_O_2_ and produce ROS, as shown in Fig. 5a. These NPs could realize the accurate release of Cu and effectively reduce biological toxicity. Moreover, the photothermal conversion ability of CuS and the catalytic effect of Cu^2+^ greatly improved the tumor inhibition ability of AIBA@CuS-FA NPs, as depicted in Fig. 5b. Similarly, Cu_2-x_S NPs (particle size less than 5 nm) also have photothermal conversion ability and the Fenton effect [61]. However, there are some big differences between Cu_2-x_S and CuS. Cu_2-x_S NPs have unpaired electrons, a large number of free carriers, and an excess of holes, which makes Cu_2-x_S NPs have the potential to be a contrast agent and the ability to produce more ROS. Moreover, Cu_2-x_S NPs have better photothermal conversion ability than CuS so that the Fenton-like reaction of Cu_2-x_S NPs in solid tumors could be better enhanced with the increasing tumor temperature. The Fenton-like reaction of Cu_2-x_S NPs in solid tumors is shown in Fig. 5c. Figure 5d indicated that the combined nano catalytic therapy (NCT)/PTT could significantly inhibit tumor growth, and the tumor in mice was completely removed after 14 days of treatment. Therefore, the life span of mice could be highly extended.

**Fig. 6:**
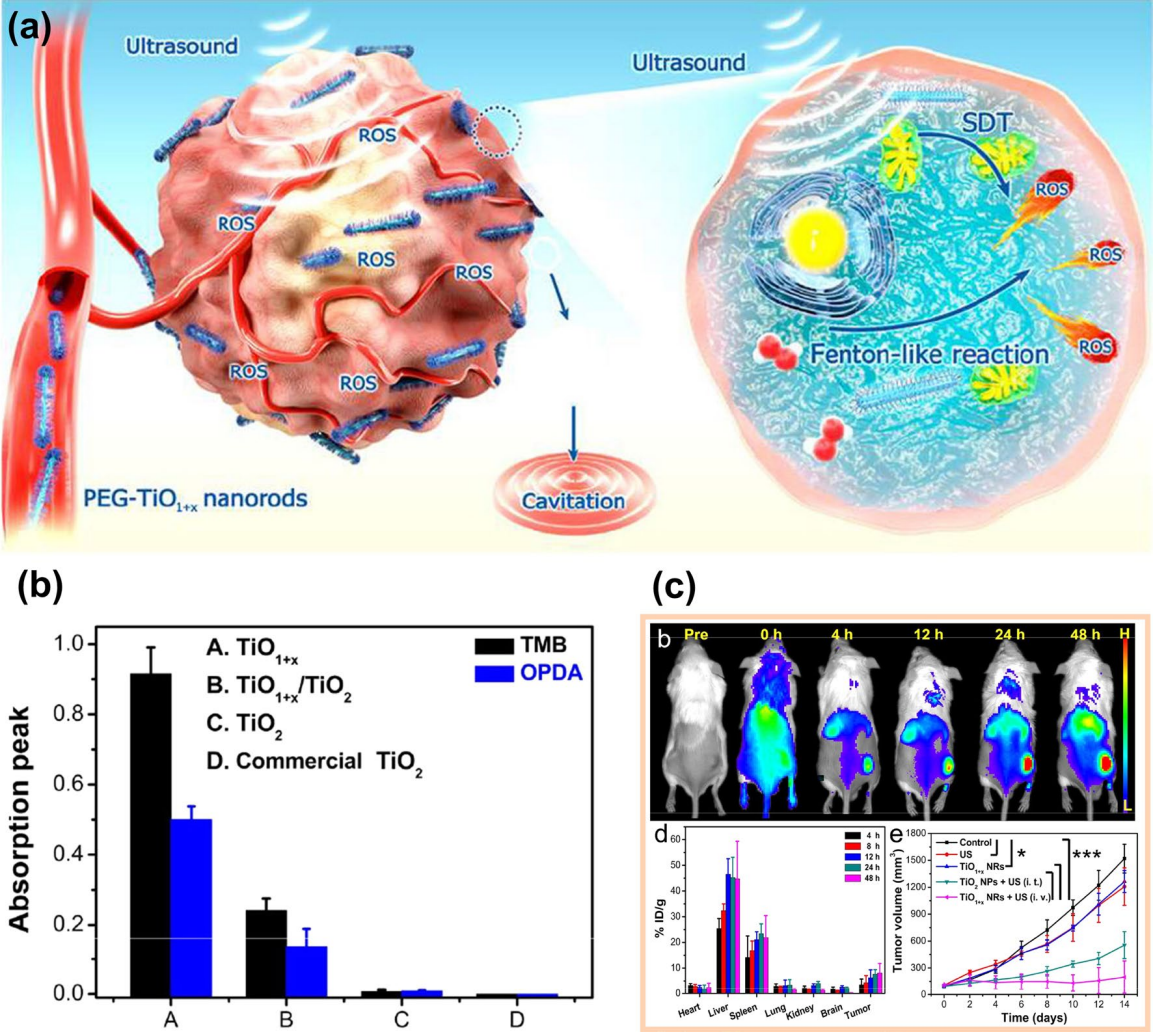
**a** Schematic diagram of ultrafine TiO_1+x_ NRs used as a novel sonosensitizer for SDT/CDT of cancer [72]. **b** CDT effects of four nanoparticles were detected by TMB and OPDA probes, respectively [72]. **c** The *in-vivo* fluorescence imaging and biodistribution of TiO_1+x_ NRs of 4T1 tumor-bearing mice after different treatment at various time points and the relative tumor volume of mice after different treatments. Reproduced with permission. [72] Copyright 2020, American Chemical Society

TiO_2_ is an inorganic sonosensitizer with high chemical stability and low phototoxicity, which is widely used in SDT. However, when pure TiO_2_ is used as a sonosensitizer, the rapid recombination of electrons and holes will reduce the quantum yield of ROS, so blocking the recombination of electrons and holes is an effective strategy to improve the production of ROS by TiO_2_ nanomaterials [71]. As mentioned above, oxygen defects can hinder the recombination of electrons and holes, so constructing TiO_2_ with oxygen defects is beneficial to improve the generation rate of ROS. More importantly, due to the existence of anoxic structures, titanium oxide compounds have a variety of valence states of titanium ions, and Ti^3+^ ions make Ti-based nanomaterials have the ability to catalyze Fenton-like reactions. For instance, the TiO_1+ x_ nanorods prepared by a typical organic phase synthesis strategy showed a high generation rate of ROS and satisfactory catalytic capacity [72]. The low bandgap of TiO_1+x_ could enhance electron–hole separation efficiency, and then the ROS generation could be improved under the action of ultrasound. In addition, there were Ti^2+^, Ti^3+^, and a small amount of Ti^4+^ in TiO_1+x_ nanorods. By comparing TiO_1+x_ nanorods with other Ti-based nanomaterials, TiO_1+x_ nanorods had the strongest catalytic performance, indicating that Ti^3+^ has a Fenton-like effect, as depicted in Fig. 6b. Moreover, the in vivo fluorescence imaging and biodistribution of PEG-TiO_1+x_ NRs in mice proved that this nanorod had good permeability and retention effect (Fig. 6c), and the tumor growth curves of different groups of mice after various treatments indicated that the therapeutic effect of TiO_1+x_ NRs was much higher than TiO_2_ NPs. Similarly, Liang et al. [23] prepared an octahedral MOF(Ti) with H_2_ as the reducing agent, the MOF(Ti) also contains the anoxic structure of TiO_x_, and the ability of Ti^3+^ to catalyze Fenton-like reaction is also verified.

In addition, accumulated evidence has indicated that Ag, Co^2+^, V^5+^, Ru^2+^, g-C_3_N_4_, and so on have the ability to catalyze the decomposition of H_2_O_2_. The specific mechanism of these nanoplatforms is summarized here, as shown in (Additional file 1: Table S3).

## Effective strategies of enhancing the anticancer efficacy of F-NCs

According to the essence of Fenton or Fenton-like reactions, the influencing factors related to Fenton or Fenton-like reactions are mainly related to the reaction environment and the catalytic properties of nanomaterials. Regulating TME is an effective strategy to improve the efficiency of Fenton or Fenton-like reactions. The major factors that can influence TME include temperature, pH, the concentration of H_2_O_2_ and GSH. More importantly, reducing F-NCs’ dependence on acidic environment is another way to improve the therapeutic effect of cancer treatment.

### Raising the temperature of solid tumors

Cancer cells can proliferate indefinitely without apoptosis under mild and appropriate conditions. They are easy to disperse and metastasize in the human body, so cancer cells have always been indestructible. However, cancer cells have a fatal weakness; that is, the heat resistance of cancer cells is poor. The temperature they can tolerate is not more than 42 °C, while normal human cells and tissues can withstand the high temperature of 46 °C [73]. Therefore, aiming at this weakness of cancer cells, it is an effective strategy to kill them by increasing the temperature of the tumor. In addition, the increasing temperature can enhance the catalytic activity of F-NCs, accelerate the decomposition rate of H_2_O_2_ and realize the synergistic enhancement of CDT. Raising tumor temperature can be achieved with microwave thermal therapy, infrared thermal therapy, ultrasonic thermal therapy, and magnetic hyperthermia therapy.

#### Microwave thermal therapy

**Fig. 7:**
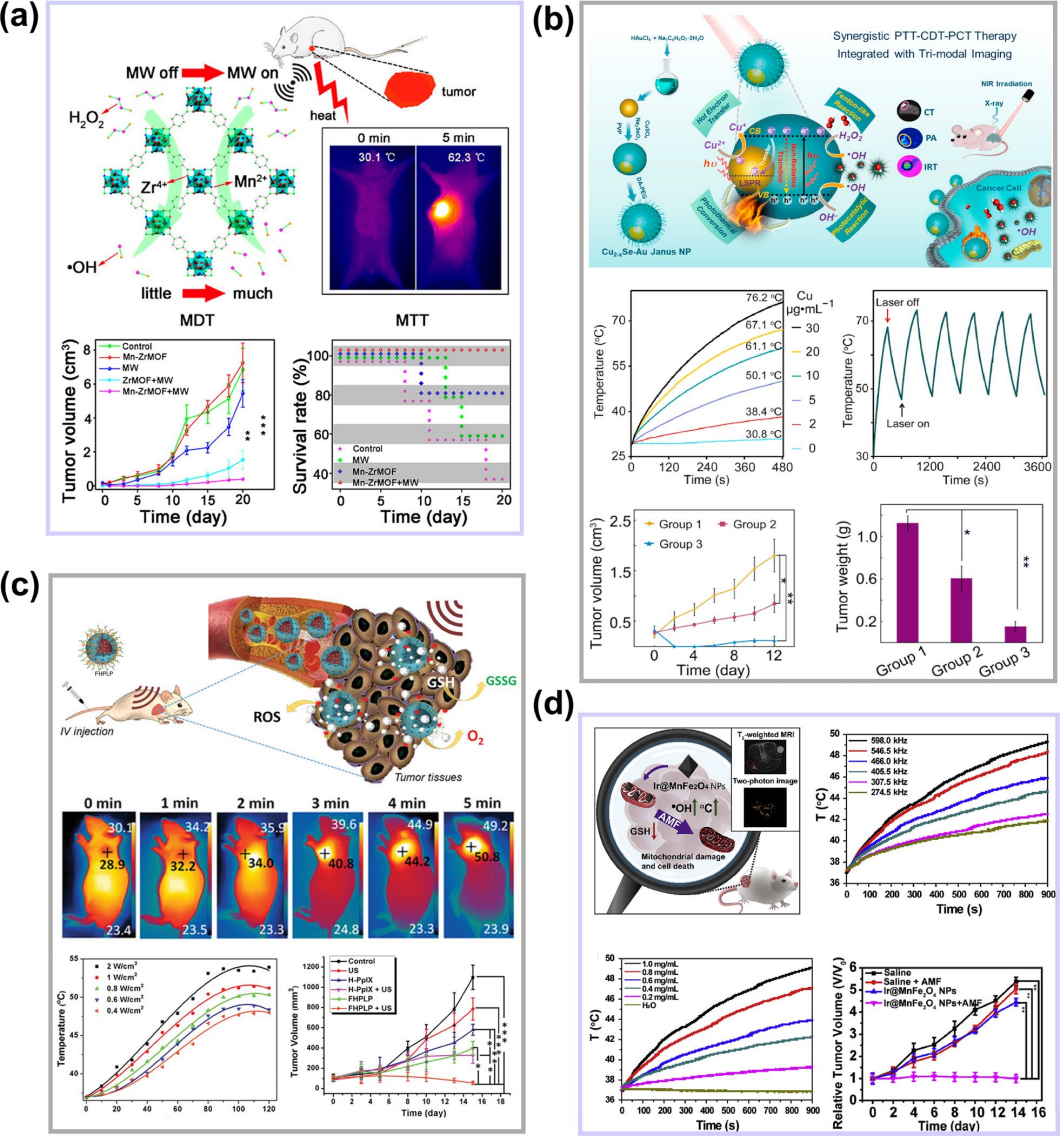
**a** The schematic diagram of Mn-ZrMOF NCs increasing tumor temperature and antitumor effect under microwave irradiation. Reproduced with permission [75] Copyright 2018, American Chemical Society. **b** Illustration of the synthesis and synergistically-combined multimodal antitumor therapies of the Cu_2-x_Se-Au Janus NPs, and the photothermal conversion effect and therapeutic effect of Cu_2-x_Se-Au Janus NPs. Reproduced with permission. [77] Copyright 2020, Elsevier Ltd. **c** The thermal and antitumor effect of FHPLP NCs under low intensity ultrasound. Reproduced with permission. [85] Copyright 2019, WILEY–VCH. **d** The magnetothermal conversion capacity and antitumor effect of Ir@MnFe_2_O_4_ NPs under AMF irradiation. Reproduced with permission. [87] Copyright 2020, Elsevier Ltd

Microwave thermal therapy (MTT) is mainly achieved by thermal effect and biological effect. Due to the existence of magnetoresistance between polar molecules, the damping effect of the oscillations consumes microwave energy and generates heat [74]. Microwave has strong penetration ability and can be used to treat deep tumors. However, due to the limited area of tumor ablation, the recurrence rate of traditional MTT is very high. Therefore, the introduction of microwave sensitizers in MTT can effectively improve the diffusion and accumulation of heat in the tumor. Moreover, if the microwave sensitizers contain some ions with Fenton or Fenton-like effect, the therapeutic efficiency of microwave sensitizers will be highly improved. Based on this theory, a new-style flexible Mn-doped zirconium metal–organic framework nanocubes (Mn-ZrMOF NCs) synthesized by Fu’s group were applied to MTT [75]. Mn-ZrMOF NCs have good microwave thermal conversion capability, and the thermal conversion efficiency is up to 28.7%. After 5 min of microwave irradiation, the temperature of tumor tissue could be raised to 62.3 °C. In addition, the catalytic efficiency of Mn^2+^ can be rapidly improved due to high temperature, which can greatly improve the anticancer effect, as shown in Fig. 7a.

#### Photothermal therapy

Infrared thermal therapy, also known as photothermal therapy, is considered to be one of the most promising treatment methods due to its low invasive and high selectivity. PTT uses photothermal conversion materials to convert light into heat to raise the temperature of the focal area and kill cancer cells. PTT combined with CDT is an effective way to improve the killing efficiency of cancer cells. Wang and co-workers [76] demonstrated that mono-dispersed CoS_2_ nanoclusters with photothermal conversion ability could significantly improve CDT. The photothermal conversion ability of CoS_2_ significantly increased the internal temperature of the tumor and the Fenton-like catalytic reaction rate. Sun et al. [77] utilized the photothermal conversion capacity and catalytic effect of Cu_2-x_Se and Au for effective antitumor therapy. The amorphous form of Cu_2-x_Se and the catalysis of Au could promote the generation of •OH. In Cu_2-x_Se-Au Janus nanoplatform, the plasmonic electrons of Au could intensify the conversion from Cu^2+^ and Cu^+^. More importantly, both Cu_2-x_Se and Au contributed to increasing the temperature (up to 67.1 °C, 30 μg/ml Cu ions) of TME under 808 nm laser, which highly improved the therapeutic effect, as depicted in Fig. 7b. In addition to Au and CoS_2_, CuS [78], Cu_9_S_5_ [79], MoS_2_ [80], and so on have also been proved to have photothermal conversion ability and can be used to increase the temperature inside the tumor and enhance the catalytic efficiency of Fenton reagent subsequently.

#### Ultrasonic thermal therapy

Ultrasound can be used for various biomedical applications; and the earliest medical application was the heating treatment of tissue. Ultrasound is divided into high-intensity focused ultrasound (HIFU) and low-intensity ultrasound. HIFU can significantly raise the temperature inside the tumor. At present, HIFU is an optional treatment method that focuses energy on deep tumor tissues in vivo but does not produce or only causes minor damage to normal tissues. Compared with PTT, HIFU can treat not only superficial tissues but also deep tissues in vivo [81], which can solve the disadvantage of insufficient light penetration in PTT. The main therapeutic mechanisms of HIFU are the thermal effect and cavitation effect [82]. The reason why HIFU has a thermal effect is that the energy of ultrasound in tissue propagation is absorbed by the tissue and converted into heat energy, which makes the tissue temperature rise. Low frequency and high energy focused ultrasound can make the temperature at the focal point rise abruptly, resulting in instantaneous high temperature (the temperature can rise 65–100 °C in 0.5–1.0 s), thus causing irreversible coagulation necrosis of the tissue [82]. Thermal ablation of tumors by HIFU has entered the clinical stage [83]. The enhancement of tumor temperature and Fenton reagent catalytic rate by combining HIFU and F-NCs should be a novel strategy [84]. In addition, high frequency and low energy focused ultrasound has also been proved to own a thermal effect and can be used to improve the catalytic efficiency of F-NCs. In recent work, the Fe(VI)@HMON-PpIX-LA-PEG NCs (FHPLP NCs) can improve the temperature (up to 50 °C) of TME after 5 min ultrasonic irradiation (1.0 MHz, 1.4 W/cm^2^) [85]. The ultrasonic thermal effect could accelerate the catalysis of Fe^2+^ and enhance the antitumor ability of this nanoplatform (Fig. 7c).

#### Magnetic hyperthermia therapy

Magnetic hyperthermia therapy (MHT) has attracted more and more attention in recent years because of its non-invasive, less damage to normal tissue, low cost, and good tissue penetration. MHT is a technology that uses magnetic nanoparticles to produce a large amount of heat to ablate tumors under a strong alternating magnetic field (AMF). These magnetic nanoparticles that can be used in MHT always are F-NCs with magnetic response-ability, such as Fe_3_O_4_, γ-Fe_2_O_3_, and so on [86]. The high temperature produced by magnetic nanoparticles can improve their catalytic capacity, realize the combined treatment MHT/CDT. For example, Ir@MnFe_2_O_4_ NPs with mitochondrial targeting properties have an obvious thermal effect under strong AMF [87]. Figure 7d indicates that the thermal effect can increase the conversion rate of Fe (III) to Fe (II) and H_2_O_2_ to •OH, and as a consequence significantly enhance the therapeutic effect of CDT.

Briefly, these four methods can be used to increase the temperature of TME and synergistically enhance the anticancer effect of F-NCs. PTT and HIFU have the most obvious effect on the increase of TME temperature, while MT, low-energy ultrasound therapy, and MHT are relatively weak. However, due to the inherent defects of PTT (such as insufficient light penetration depth), the temperature of PTT for deep tumors will be seriously limited, and the instantaneous heating effect of HIFU will greatly improve its operation difficulty. Therefore, using low-energy ultrasound and AMF with high penetration depth to enhance the temperature of deep TME is a good choice. However, it is worth noting that in order to achieve the ideal treatment temperature, the concentration of materials used in MHT is generally higher than that of the other three therapies, which may increase the potential toxicity of this therapy. Therefore, materials with higher magnetocaloric conversion ability need to be developed urgently.

### Reducing the pH of the TME and the acid dependence of F-NCs

**Fig. 8:**
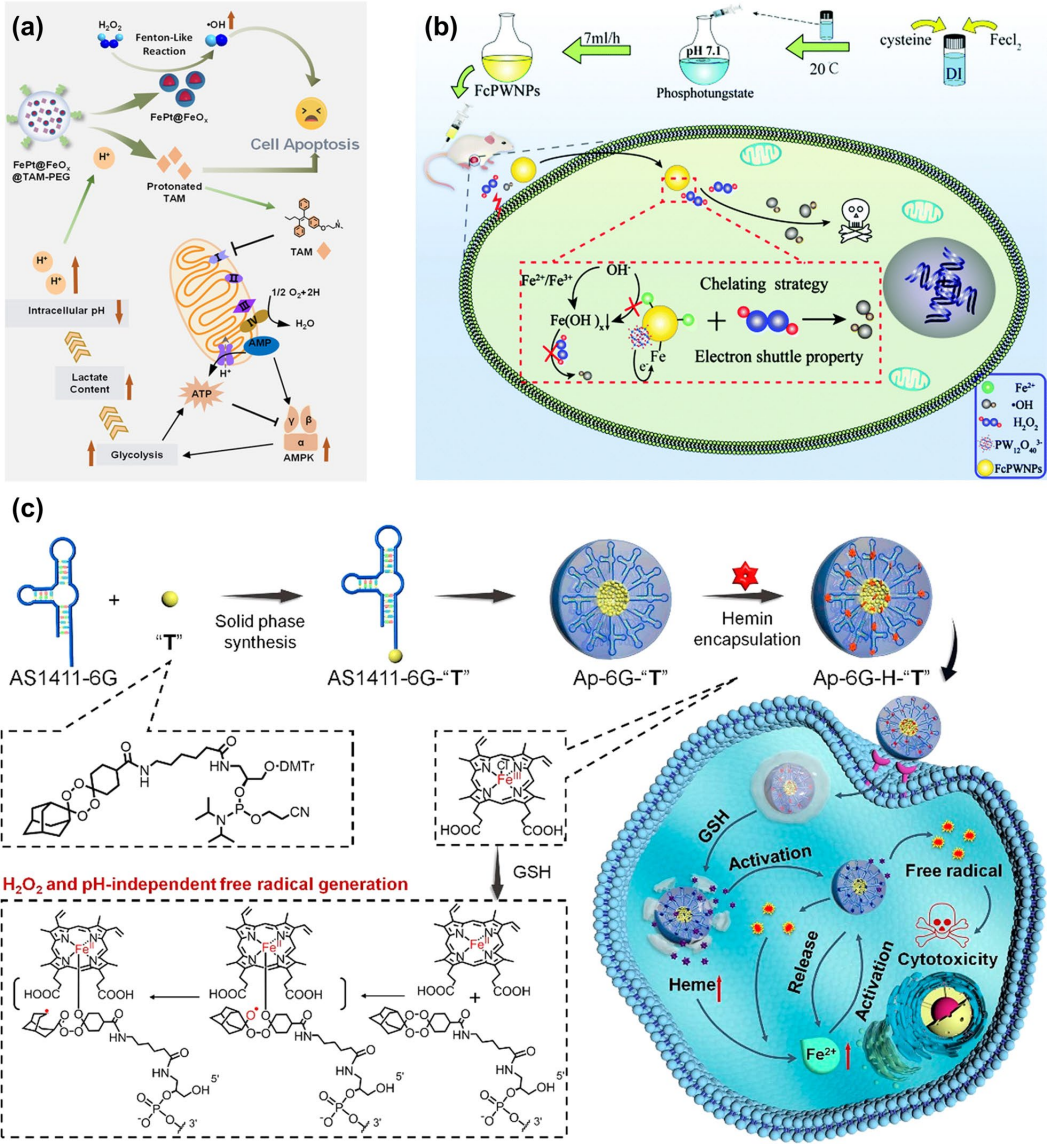
**a** Specific process of FePt@FeOx @TAM-PEG regulating pH in tumor cells. Reproduced with permission. [93] Copyright 2021, Wiley–VCH. **b** The mechanism of FcPWNPs mediated efficient CDT without acid dependence. Reproduced with permission. [94] Copyright Clearance Center, Inc. **c** Synthesis of aptamer prodrug conjugate ApDC nanoplatform and its mechanism in tumor. Reproduced with permission [95]. Copyright 2019, American Chemical Society

The pH in TME is 6.5–7, while the optimal pH for the Fenton reaction is 2–4 [88]. Therefore, reducing the pH of TME is also an effective choice to improve the efficiency of the Fenton reaction [89]. Generally, increasing the acidity of the TME can be achieved by introducing exogenous acids or other substances that can regulate the pH of the TME. As mentioned above, DCA is a strong organic acid with a pKa value of 1.35, which can regulate the pH of TME. It has been widely used in the clinical treatment of cancer [90–92]. The addition of DCA can not only reduce the pH of TME but also can reduce the mitochondrial membrane potential and greatly increase the reaction rate of the Fenton reaction. In addition to introducing DCA, introducing tamoxifen (TAM) has also been proved to be able to regulate the pH of the TME. However, different from DCA, TAM can indirectly regulate the pH of TME. For example, Shi et al. [93] synthesized a pH-responsive nanoplatform (FePt@FeO_x_@TAM-PEG). TAM is an anti-estrogen drug that can inhibit mitochondrial complex I, resulting in an increase in the ratio of adenosine monophosphate (AMP) to adenosine triphosphate (ATP), which can trigger the AMP-activated protein kinase (AMPK) signaling pathway. AMPK is a major factor that could regulate energy homeostasis in cells, which can promote glucose decomposition and lactic acid accumulation subsequently and increase the acidity in cancer cells finally, as shown in Fig. 8a. The increase of intracellular acidity can accelerate the release of FePt@FeO_x_ NPs, thereby releasing Fe^2+^ and Fe^3+^ ions, accelerating the decomposition rate of H_2_O_2_, and enhancing the anti-tumor ability of the nanoplatforms.

Introducing exogenous acids or substances that can reduce the pH of tumors is a common and effective means to regulate the pH of TME. But it is a difficult issue to control the dosage of exogenous acid or other chemicals with pH-adjustable properties. Therefore, to solve this problem, the development of Fenton reagents without acid dependence is also an effective strategy to increase the Fenton reaction rate. The low efficiency of Fenton reaction-mediated CDT is due to the rapid precipitation of iron ions into inert Fe(OH)_x_ in tumor tissues (pH 6.5–7) and the slow conversion rate between Fe^3+^ and Fe^2+^. The reduced concentration of Fe^2+^ seriously affects the reaction rate of the Fenton reaction. The excessive dependence of Fenton reagent on the acidic environment can be solved by blocking the transformation of iron ions to inert Fe(OH)_x_. For example, the cysteine-iron phosphotungstate chelate NPs (FcPWNPs) could avoid the transformation of iron ions to Fe(OH)_x_ to break the limitation of pH [94]. The electron shuttle property of phosphotungstate can accelerate the transfer of Fe^3+^ to Fe^2+^, and the chaining Fe^2+^ will not transform into inert Fe(OH)_x_ under neutral conditions, so that FcPWNPs can produce •OH on the neutral surface and in the acidic interior of the tumor (Fig. 8b). Moreover, because of the excess mitochondrial metabolism, the concentration of H_2_O_2_ in tumor cells is several hundred mM, which is much higher than that in normal cells. Using prodrugs to reduce the acid dependence of Fenton reagents is also a way to increase the activity of Fenton reagents. For instance, a novel apder-prodrug conjugate (ApDC) could be used for non-H_2_O_2_ and pH-dependent CDT [95]. ApDC micelle is mainly composed of three parts. The first one is the aptamer that identifies cancer cells; the second one is the prodrug base of the Fe^2+^ activated tetraoxane (T); the third one is the heme that could respond to TME and provide Fe^2+^ for in situ activations of T. Unlike conventional micelles, the prodrug in these micelles contains hydrophobic prodrug bases that not only promote aptamer assembly but form many free radicals through bioorthogonal reactions. More importantly, the strong hydrophobic prodrug bases could achieve the loading of heme in the ApDC micelles and improve the targeting ability of the aptamer-prodrug conjugate (ApPdC) micelle to the nucleus. As the number of “T” bases in a single ApPdc chain increased to three, the non-specific binding of ApPdC micelles to HepG2 cells became very apparent. In this nanoplatform, the free radical production process is independent of strong acidity or endogenous H_2_O_2_ and simultaneously weakens the antioxidant capacity of cancer cells by consuming GSH. Although the cytotoxicity of this nanoplatform does not come from hydroxyl radicals. It depends on the C-centered toxic free radicals, and the production of the C-centered poisonous free radicals relies on the concentration of Fe^2+^ ions, as shown in Fig. 8c. Therefore, designing the prodrug can fundamentally solve the issue of the conversion of Fe^2+^ into Fe(OH)_x_ in a neutral environment.

According to the above analyses, increasing the reactivity of Fenton reagents can start from reducing the pH of the TME by introducing exogenous acids or chemicals that can regulate the pH value of the TME. However, more importantly, the preparation of Fenton reagents that can be independent of the acidic environment has a wider application prospect and will be a new research trend of Fenton reagents in the future.

### Increasing the concentration of H_2_O_2_ in TME

H_2_O_2_ is one of the reaction substrates of Fenton or Fenton-like reactions. The concentration of H_2_O_2_ in the tumor is 100 μM, five times higher than normal cells [96]. However, this concentration still fails to achieve the ideal effect for cancer therapy. Therefore, increasing the concentration of H_2_O_2_ can enhance the efficiency of the Fenton reaction and the anti-cancer therapeutic effect of Fenton reagents. Here, three methods to increase the concentration of H_2_O_2_ in TME are summarized, including adding exogenous H_2_O_2_, some chemical agents that can enhance the level of endogenous H_2_O_2_, and metal peroxides to improve the concentration of H_2_O_2_ synergistically.

#### Addition of exogenous H_2_O_2_

**Fig. 9:**
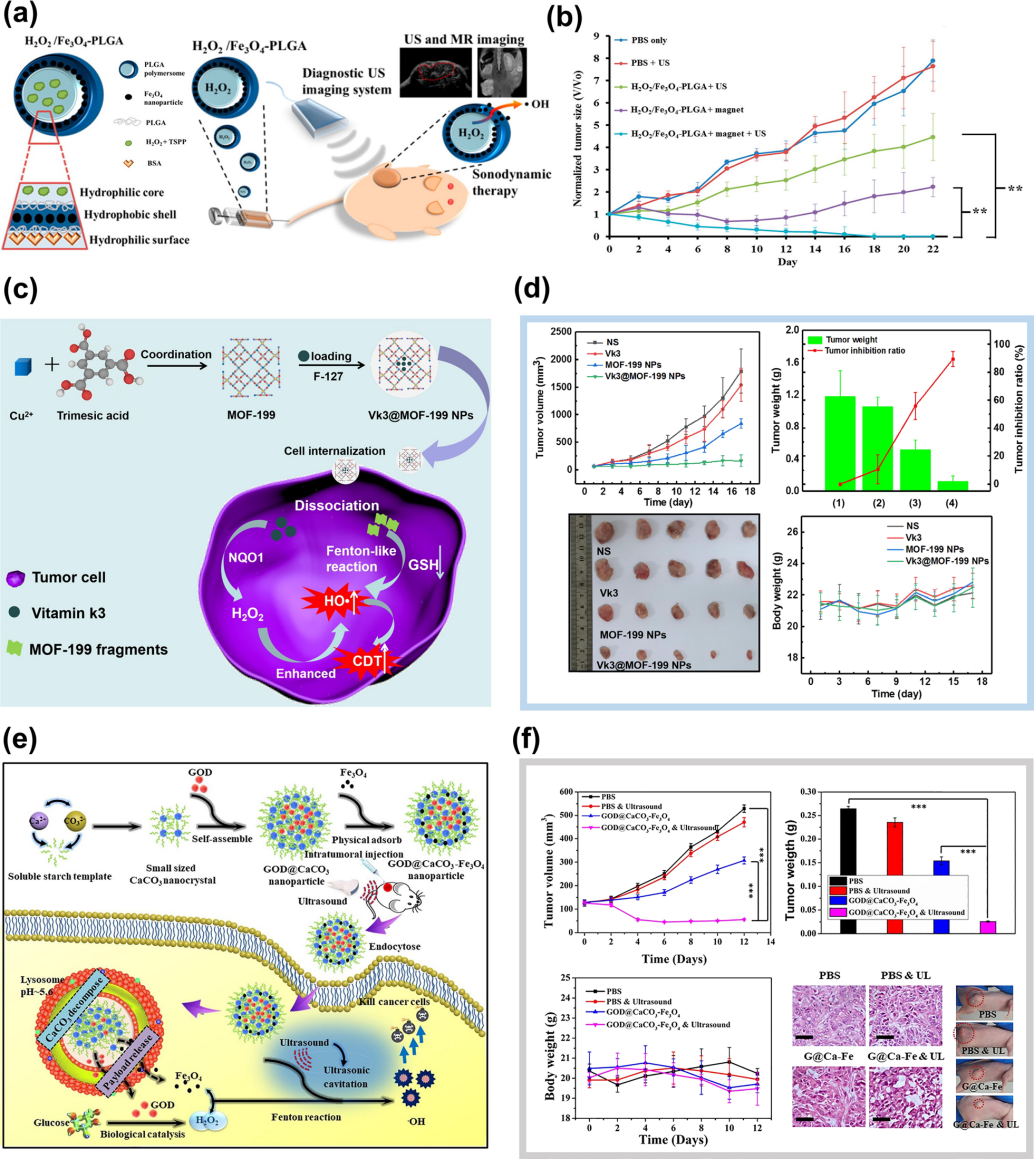
**a** The structure and action mechanism of H_2_O_2_/Fe_3_O_4_-PLGA NPs [88]. **b** Tumor growth curves with different treatments (n = 3) after intravenous injection (**p < 0.01). Reproduced with permission. [97] Copyright 2016, American Chemical Society. **c** Illustration of the synthesis and mechanism of Vk3@MOF-199 NPs [96]. **d** The tumor volume, tumor weight, tumor photos, and bodyweight of mice after different treatments. Reproduced with permission. [96] Copyright 2020, Elsevier Inc. e The synthetic procedure for GOD@CaCO_3_-Fe_3_O_4_ particles and the degradation and action mechanism of Fe_3_O_4_@GOD@CaCO_3_ NPs in the tumor [103]. **f** In vivo anticancer effect of GOD@CaCO_3_-Fe_3_O_4_ particles assisted by ultrasound irradiation. Reproduced with permission. [103] Copyright 2019, Elsevier B.V

H_2_O_2_ is the reaction substrate of Fenton or Fenton-like reactions, so the introduction of exogenous H_2_O_2_ is the most direct way to improve the Fenton reaction rate. H_2_O_2_ is unstable, and it will decompose quickly at room temperature. To introduce exogenous H_2_O_2_ in cancer cells directly, liquid H_2_O_2_ encapsulated in the hydrophilic cores of NPs to improve the concentration of H_2_O_2_ in TME will be a good choice. For example, the H_2_O_2_/Fe_3_O_4_-PLGA nanoplatforms could directly transport liquid H_2_O_2_ to the tumor [97]. In this system, liquid H_2_O_2_ and disodium triphosphate were encapsulated in a hydrophilic core in the first emulsification, and Fe_3_O_4_ NPs were embedded into a PLGA polymer hydrophobic shell in the second emulsification process. The loading amount of H_2_O_2_ could be realized by adjusting the concentration of H_2_O_2_ in the first emulsion polymerization and did not affect the morphology and size of NPs. Iron oxide mainly existed in the PLGA polymer shell. The encapsulation of H_2_O_2_ played a key role in the treatment process. It could not only produce O_2_ for echo reflection to achieve ultrasound imaging but also provide reaction substrate H_2_O_2_ for the Fenton reaction (Fig. 9a). The interaction between H_2_O_2_ and iron ions greatly increased the level of ROS in the TME, and the tumor in mice was significantly inhibited (Fig. 9b). Similarly, Song et al. [98] also solved the hypoxia in a tumor by introducing exogenous H_2_O_2_. They encapsulated catalase (CAT) and H_2_O_2_ in liposomes to obtain CAT@Liposome and H_2_O_2_@Liposome NPs, respectively. They found that the combination of CAT@Liposome and H_2_O_2_@Liposome could significantly improve the effect of cancer treatment. CAT can promote the decomposition of H_2_O_2_ to produce ROS, which highly improves the concentration of ROS in cancer cells.

#### Introduction of chemical agents

Increasing the concentration of H_2_O_2_ in TME by introducing exogenous chemical agents is the most widely used research method at present. GOD, doxorubicin (DOX), cinnamaldehyde (CA), vitamin k3(Vk3), β-lapachone (Lap), and some other exogenous chemicals have been found to increase the concentration of H_2_O_2_ in tumors [99, 100]. Tian and co-workers [96] designed a Cu-based MOF-199 nanoplatform integrated with Vk3. The nanoplatform could dissociate into MOF-199 fragments by reacting with GSH in the tumor and release Vk3 that could be catalyzed by NAD(P)H quinone oxidoreductase-1(NQO1) to produce enough H_2_O_2_ to activate the Fenton-like reaction, as shown in Fig. 9c. The lightest and smallest tumors of the Vk3@MOF-199NPs group indicated that Vk3 could synergistically enhance CDT in vivo under the action of the NQO1 enzyme, as depicted in Fig. 9d. In addition, the use of GOD to increase the concentration of H_2_O_2_ in TME is also an effective method [100–103]. GOD can oxidize glucose in tumor cells to H_2_O_2_, which can continuously provide an oxygen source for tumor treatment. Glucose in cells is oxidized and consumed, making cancer cells lack nutrients and starve, so this treatment process is also known as "Starvation therapy" [104]. Chen et al. [103] synthesized Fe_3_O_4_@GOD@CaCO_3_ NPs with the Fenton effect. Under the effect of the template of soluble starch, the nanocrystalline generated by the reaction of Ca^2+^ and CO_3_^2−^ ions immediately self-assembled into CaCO_3_ nanocrystals. GOD was added before the completion of self-assembly to ensure the loading of GOD into the interior of CaCO_3_ nanocrystals to obtain GOD@CaCO_3_ NPs, and then the Fe_3_O_4_ NPs prepared by the thermal solvent method were adsorbed on GOD@CaCO_3_ NPs by physical adsorption. Finally, the Fe_3_O_4_@GOD@CaCO_3_ NPs with the Fenton effect was obtained, as shown in Fig. 9e. The advantage of this nanoplatform is that CaCO_3_ and Fe_3_O_4_ can degrade in TME to produce Ca^2+^ and Fe^2+^. They are essential trace elements in the body so that low dose Fe_3_O_4_@GOD@CaCO_3_ NPs have low bio-toxic to mice. Due to the degradation of this nanoplatform, GOD could be successfully released into TME and oxidize glucose in the tumor to produce H_2_O_2_, resulting in a rich oxygen environment for tumor therapy. Under the effect of ultrasound, Fe^2+^ could catalyze the decomposition of H_2_O_2_ to produce ROS and couple with the overload of Ca^2+^, greatly increasing the apoptosis rate of cancer cells, as shown in Fig. 9f.

#### Introduction of metal peroxides

Metal peroxides can produce H_2_O_2_ through a disproportionation reaction with water and can generate a strong oxidation effect through decomposition products (such as H_2_O_2_) under acidic conditions [105, 106]. Meanwhile, they can slowly release O_2_ in water or under heating conditions. Therefore, using metal peroxides is also a new method to increase the concentration of H_2_O_2_ in tumors [107].

**Fig. 10:**
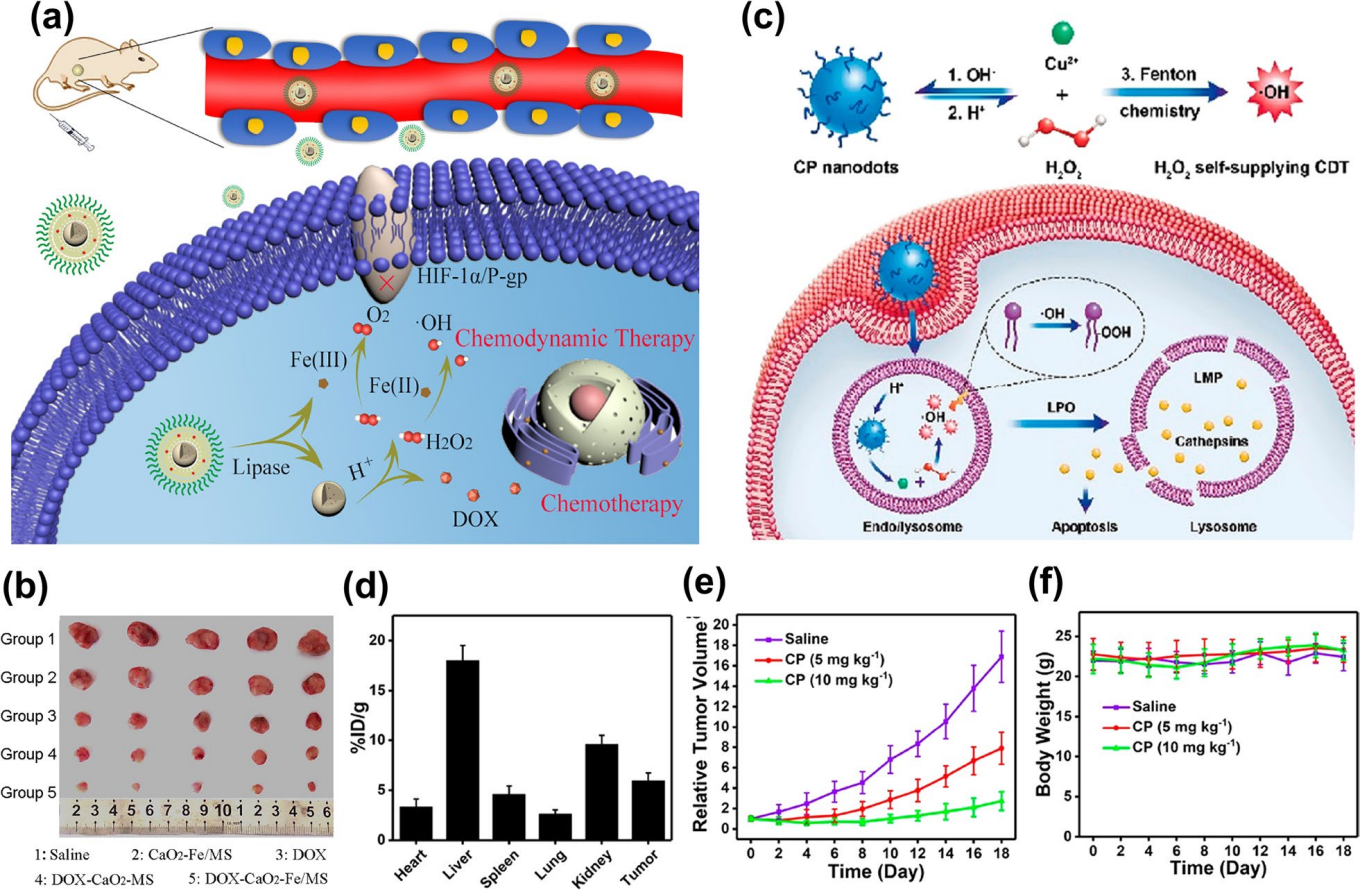
**a** Schematic diagram of the anticancer mechanism of DOX-CaO_2_-Fe/MS nanoplatform [110]. **b** Tumor photographs of mice treated by different groups. Reproduced with permission. [110] Copyright 2020, Acta Materialia Inc. **c** CP nanoparticles enhance the anticancer effect by increasing the level of H_2_O_2_ in the tumor [111]. **d** Biodistribution of Cu in major organs and tumor of U87MG tumor-bearing mice at 24 h post i.v. injection with CPs [111]. **e** Relative tumor growth curves of U87MG tumor-bearing mice after treatment with saline (control group) or different doses of CPs [111]. **f** Time-dependent body-weight curves of mice in different groups. Reproduced with permission. [111] Copyright 2019, American Chemical Society

At present, the metal peroxide mainly used in cancer treatment is CaO_2_. Moreover, ZnO_2_, MgO_2_, BaO_2_, CuO_2_, etc*.*, have also been found to be able to increase the concentration of H_2_O_2_ in tumors [107–109]. In order to achieve self-sufficiency of O_2_/H_2_O_2_ in the tumor, He et al. [110] synthesized a DOX-CaO_2_-Fe nanoplatform containing chemotherapy drug DOX and biocompatible Fenton catalyst ferric oleate complex. Because of the easy decomposition of CaO_2_ in water and acidic environment, solid lipid monostearate was used to coat CaO_2_ to avoid the premature decomposition of CaO_2_. In the body, the overexpression of lipase can degrade the lipid layer of NPs. CaO_2_ can be exposed to the acidic microenvironment of the tumor and react with the acidic water environment to produce H_2_O_2_. Finally, the chemotherapy drug DOX and ferric oleate will be released. Fe^3+^ in ferric oleate could react with H_2_O_2_ to produce O_2_ and Fe^2+^, and Fe^2+^ could catalyze H_2_O_2_ to generate ROS. The anticancer mechanism of the DOX-CaO_2_-Fe/MS nanoplatform is shown in Fig. 10a. In vivo experiments showed that the addition of CaO_2_ endowed the nano platform with the ability to solve the problem of hypoxia in TME. The synergistic effect between CaO_2_ and Fe could obviously increase the tumor inhibitory effect of DOX-CaO_2_-Fe/MS NPs (Fig. 10b). Different from CaO_2_, CuO_2_ can not only enhance H_2_O_2_ levels in tumors but also act as a Fenton catalyst for hydrogen peroxide decomposition, such as copper peroxide nano points (CP) [111]. The CP nanoplatform was synthesized by the coordination of H_2_O_2_ and Cu^2+^ with the help of hydroxide ions. After the CPs enter tumor cells, the acidic environment of lysosomes would accelerate the degradation of CPs and produce H_2_O_2_ and Cu^2+^ at the same time, and then Cu^2+^ catalyzed the decomposition of H_2_O_2_, as shown in Fig. 10c. Due to the small hydrodynamic diameter (16.3 nm) of CPs, they could take advantage of the EPR effects to efficiently accumulate in tumors. By evaluating the biological distribution of CPs in U87MG tumor-bearing mice, the uptake rate of CPs by tumor reached 5.96 ± 0.79%ID/g after 24 h of intravenous injection (Fig. 10d). The high accumulation of CPs in vivo made it suitable for in vivo CDT without obvious side effects. More importantly, the ability of CPs to generate H_2_O_2_ in vivo could significantly improve the tumor inhibition effect of CDT, as depicted in Fig. 10e, f.

### Reducing the level of GSH in tumor cells

GSH is a tripeptide composed of glutamate, cysteine, and glycine that can be found in almost every cell in the human body [112, 113]. GSH has antioxidant properties and integrates detoxification to remove free radicals in the human body. GSH is easily oxidized by free radicals with oxidative properties in the body and the free radicals can be reduced to acidic substances, so as to accelerate the excretion of free radicals and reduce the toxic and side effects of free radicals on normal cells. In addition, the strong reducibility of GSH can protect the sulfhydryl groups in important enzyme proteins in normal cells from oxidation and inactivation, so as to ensure the metabolism of normal cells [114]. However, overexpressed-GSH in tumors will decrease the level of ROS in cancer cells. Therefore, the therapeutic effect can be improved by reducing the level of GSH for cancer cells.

**Fig. 11:**
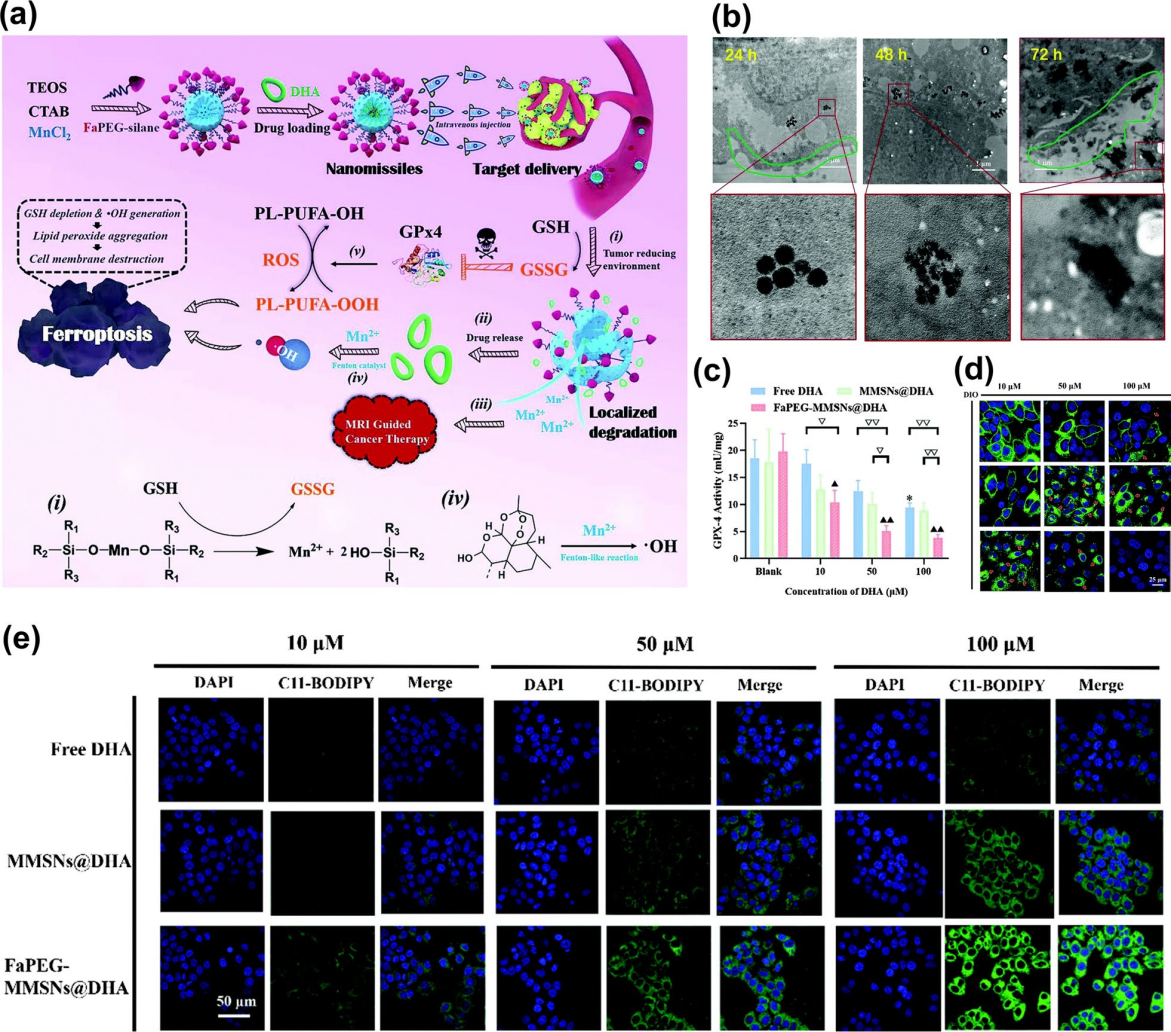
**a** Schematic illustrations of the construction and ferroptosis-inducing mechanism of FaPEG-MMSNs@DHA nano missiles [115]. **b** Intracellular biodegradation behavior of FaPEG-MMSNs@DHA [115]. **c** GPx4 activity of HepG2 cells after different treatments [115]. **d** The membrane morphological changes of HepG2 cells stained with DIO after co-incubation with different groups [115]. **e** PL-PUFA-OOH detection assay of HepG2 cells stained with C11-BODIPY^581/591^ after co-incubation with different groups. Reproduced with permission [115] Copyright Clearance Center, Inc

Enhancing the anticancer effects of Fenton reagents by lowering the concentration of GSH has been proved to be a viable strategy. For example, a mesoporous silica nanosystem (FaPEG-MMSNs@DHA) constructed by Fei’s group was used to deplete GSH and enhance ROS production, which was modified by folate-polyethylene glycol, loaded with dihydroartemisinin (DHA), and doped with Mn [115]. When the NPs were phagocytosed by tumor cells, the Mn–O bonds in the NPs would undergo a redox reaction with GSH, and DHA and Mn^2+^ with Fenton catalytic effect could be released (Fig. 11a). On the one hand, the degradation of NPs resulted in the decrease of the level of GSH in TME, which inhibited the activity of GP_X_4, so that the ability of ROS to oxidize PL-PUFA-OH will be enhanced, leading to the accumulation of lipid peroxides (PL-PUFA-OOH) in the tumor, as shown in Fig. 11b, c, and e. In addition, the released Mn^2+^ will catalyze the reaction of peroxide bridge structure in DHA and produce •OH. The membrane morphological changes of HepG2 cells shown in Fig. 11d indicated that the cooperative effects could suppress tumor metastasis by destroying the structure of polyunsaturated fatty acids in the cell membranes and showed a potent antitumor effect. Similarly, some researchers [116–118] have synthesized organic–inorganic hybrid hollow mesoporous silica (HMONs) by the chemical homology method. HMONs have a unique nanostructure and composition. 4,4,13,13-tetraethoxy-3,14-dioxa-8,9-dichiath-4,13-disilahxadecane (BTDS), as a structural crosslinking agent, was covalently hybridized into the HMONs framework. The disulfide bond (S–S) enabled HMONs to consume reduced-GSH and be biodegradable. Therefore, HMONs have a broad application prospect in anticancer drug delivery.

### Applications of F-NCs in anticancer therapy

**Fig. 12:**
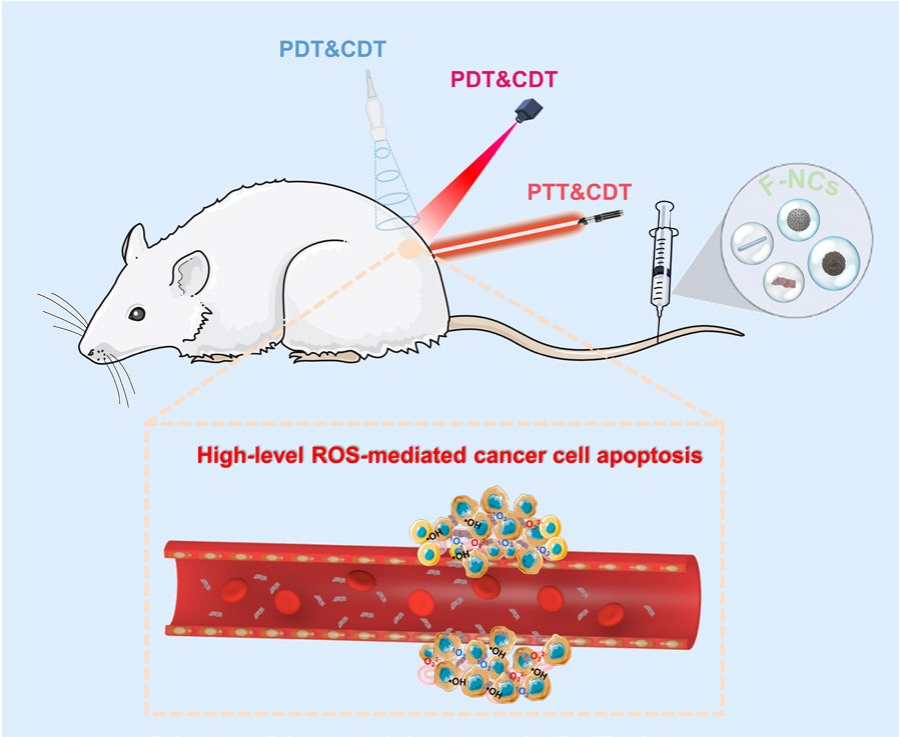
F-NCs for CDT-involved combination therapies

CDT is a classical therapy mediated by Fenton or Fenton-like reactions, which is considered a green and efficient treatment method. However, the application of CDT has been severely hampered by the shortcomings of insufficient H_2_O_2_ content in tumors to produce continuous ROS and the fact that TME is not the optimal reaction condition for F-NCs. Nowadays, most of the F-NCs are commonly combined with other treatment methods to overcome these shortcomings. More importantly, the combination of CDT with other treatments can significantly improve the efficacy of anticancer therapy. At present, the treatment methods that can be combined with CDT mainly include PTT, PDT, SDT, ST, Gas therapy, and so on. As two common external excitation energies, light and ultrasound have the unique advantages of controllable wavelength and intensity and adjustable action area. In addition, the relatively low cost and noninvasive therapy make F-NCs widely used in light-excited or ultrasound-excited cancer treatment (such as PTT, PDT, and SDT), as shown in Fig. 12 [119–121]. The applications of F-NCs in PTT, PDT, and SDT will be introduced in detail in the following subsections.

### Applications of F-NCs in PTT

As mentioned above, PTT has made huge progress in anticancer treatment due to its unique advantages. [122–124]. For example, Liu et al.have designed the NIR-light responsive and injectable DNA-mediated upconversion and gold NPs hybrid hydrogels (DNA-UCNP-Au) [125] for the treatment of the targeted treatment of malignant tumors. Xu et al.prepared the NIR-II photothermally activatable semiconducting polymeric nanoantagonist (ASPA) [126]. And Zhou et al. fabricated an activatable NIR-II plasmonic theranostics system based on silica-encapsulated self-assembled gold nano chains (AuNCs@SiO_2_) [127] for efficient photoacoustic imaging and photothermal cancer therapy. Generally, the single-mode therapy of PTT is unable to eradicate tumor cells [128]. Interestingly, some studies have shown that PTAs with Fenton or Fenton-like effects can highly improve the anticancer therapeutic effect of them.

For example, two-dimensional (2D) nanosheets (FePS_3_ NSs) with good biocompatibility and satisfactory Fenton effect could effectively eradicate tumors in mice[129]. The photothermal conversion efficiency of these NSs was 43.3%. Under the combined effect of PTT and CDT, the tumor inhibition rate was 95% after treatment with a drug concentration of 24 μg/ml. The tumor could be effectively eradicated after intravenous administration without any signs of recurrence. Similarly, another 2D nanomaterial (CuFe_2_S_3_) also displayed a highly therapeutic effect due to its excellent photothermal conversion ability and catalytic capacity[130]. The near-infrared photothermal conversion efficiency of CuFe_2_S_3_-PEG was 55.86%, resulting in a great improvement of temperature in TME. It could accelerate the reaction rate of CuFe_2_S_3_-PEG and GSH and further enhance the catalytic decomposition of H_2_O_2_ by Fe^2+^ and Cu^+^. PTT combined with CDT can eventually induce apoptosis of 87.97% hepatoma cells and complete tumor resection in vivo.

**Fig. 13:**
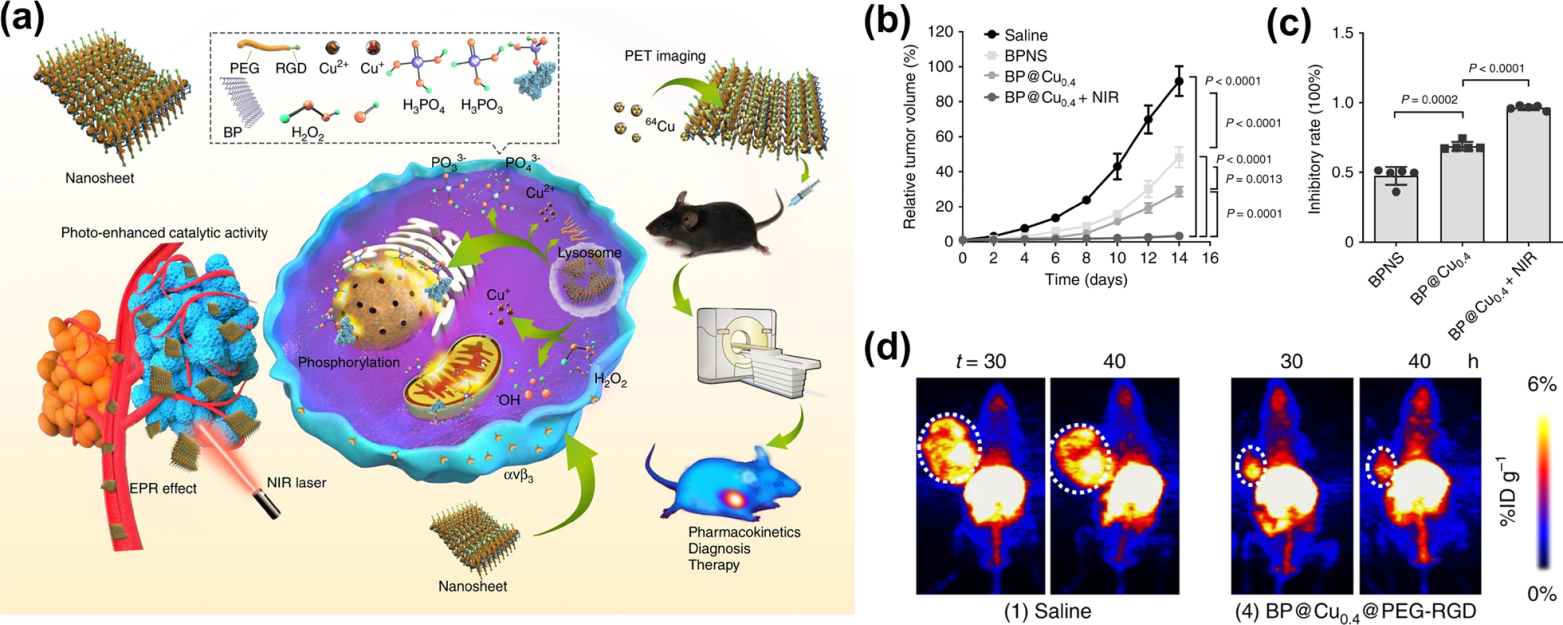
**a** Schematic diagram of the anticancer mechanism of multifunctional BP@Cu@PEG-RGD hybrid nanomaterials in vivo [133]. **b** Relative tumor volume after different treatments on day 14 [133]. **c** Inhibitory rates of B16F10 tumors at day 14 post-treatment [133]. **d** MIP PET images of B16F10 tumor-bearing mice after 2 weeks of treatment with saline or BP@Cu_0.4_@PEG-RGD, respectively. PET images were taken at 30 and 40 h after intravenous injection of saline or BP@^64^Cu@PEG-RGD. The white circles denote the tumor sites. Reproduced with permission. [133] Copyright 2020, The Author(s)

There is another advantage about PTAs with Fenton or Fenton-like effects. Sometimes, there will be a conflict between the high photothermal conversion efficiency and the degradability of photothermal agents (PTAs) [131]. PTAs with high photothermal conversion ability are difficult to degrade in vivo. This conflict will seriously limit the application of PTAs in cancer treatment. PTAs with Fenton or Fenton-like effects can solve the conflict between photothermal conversion efficiency and biodegradability. Because PTAs with catalytic ability generally can respond to the TME, which makes them own slow-degradability and little loss photothermal conversion capability. Based on this theory, Hu and co-workers [132] exploited the electrostatic attraction and coordination effect of two-dimension(2D) material black phosphorus nanosheets (BPNS) to capture Cu^2+^ and synthesized a photosensitizer (BP@Cu) with high photothermal conversion efficiency and Fenton effect that can be degradable in vivo (Fig. 13a). The coordination between Cu^2+^ and BP could enhance the degradation of BP, the thickness of BPNS@Cu is significantly lower than that of pure BPNS. In addition, the best therapeutic effect of the BP@Cu_0.4_@PEG-RGD group, as shown in Fig. 13b–d, indicated that the photothermal effect of Cu^2+^ and BPNS could synergistically enhance the CDT efficiency of Cu ions.

### Applications of F-NCs in PDT

In addition to PTT, PDT is another light-mediated cancer treatment. PDT is a type of photoactivated chemotherapy. The photosensitizer absorbs the energy of photons to the excited state and generates some oxidation active molecules. After the photosensitizer (oxidation active molecules) is injected into the body for a period of time, it will specifically gather in the tumor site and combine with tumor cells, and then the photochemical reaction will be generated after the laser irradiation with a specific wavelength [133]. Compared with PTT, PDT has irreplaceable advantages. The local high fever of tumors caused by PTT may damage normal tissues. However, PDT can convert molecular oxygen into cytotoxic ROS, which causes irreversible damage to tumors. More importantly, PDT can accurately and mildly inhibit tumor growth for a long time [128]. But the single-mode of PDT is still cannot completely kill cancer cells, so it is necessary to develop multi-mode therapy. The combination of PDT and CDT can highly improve the efficiency of PDT.

**Fig. 14:**
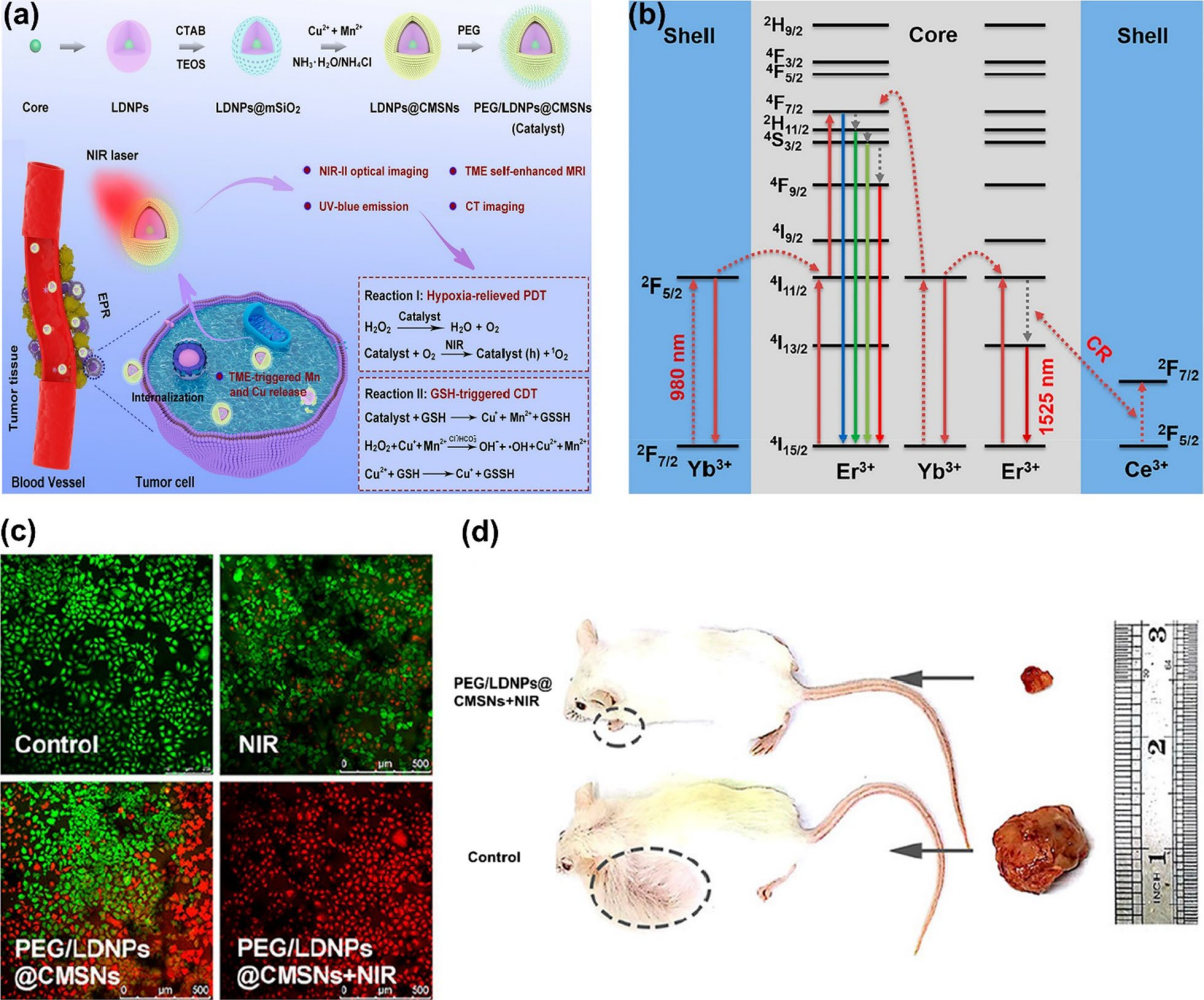
**a** The synthesis of PEG/LDNPs@CMSNs nanoplatform and its action mechanism in vivo [135]. **b** The UC and DC mechanism of LDNPs [135]. **c** Corresponding CLSM images dyed with AM and PI of HeLa cells in four groups [135]. **d** Relative tumor volume in the control group and PEG/LDNPs@CMSNs + NIR group. Reproduced with permission [135]. Copyright 2020, American Chemical Society

High levels of GSH and low concentrations of H_2_O_2_ in cancer cells are two major obstacles in PDT and CDT. In order to combine PDT and CDT with good therapeutic effects, there is an urgent need to develop Fenton effect photosensitizers that can reduce intracellular GSH levels or increase intracellular H_2_O_2_ levels. Xu et al. [134] synthesized biodegradable lanthanide-doped NPs (LDNPs) encapsulated in copper/manganese silicate nanospheres (CMSN). The doping of Yb^3+^/Er^3+^/Tm^3+^ in LDNPs endowed them the function of near-infrared laser upconversion (UC) and downconversion (DC), as shown in Fig. 14a, b. LDNPs with high atomic coefficients and unpaired electrons could be used in MRI and CT imaging. The CMSN shell could be excited by the emitted short-wavelength photons to realize PDT. It was also able to react with GSH in the tumor, resulting in the degradation of the CMSN shell and releasing Cu^+^ and Mn^2+^ Fenton-like ions, which could increase the level of •OH. There is another way to increase ROS levels in tumors about these NCs. When the up-conversion photon is activated by the NIR laser, O_2_ could react with samples to produce ^1^O_2_. Therefore, this nanoplatform solved the problems of low concentration of H_2_O_2_ and high concentration of GSH in TME to improve PDT, as shown in Fig. 14a. Figure 14c, d showed that PEG/LDNPs@CMSBNs + NIR group was more effective than other groups. The tumor was almost completely removed from the mice after the treatment of CDT/PDT, indicating Fenton NCs can dramatically enhance PDT.

**Fig. 15:**
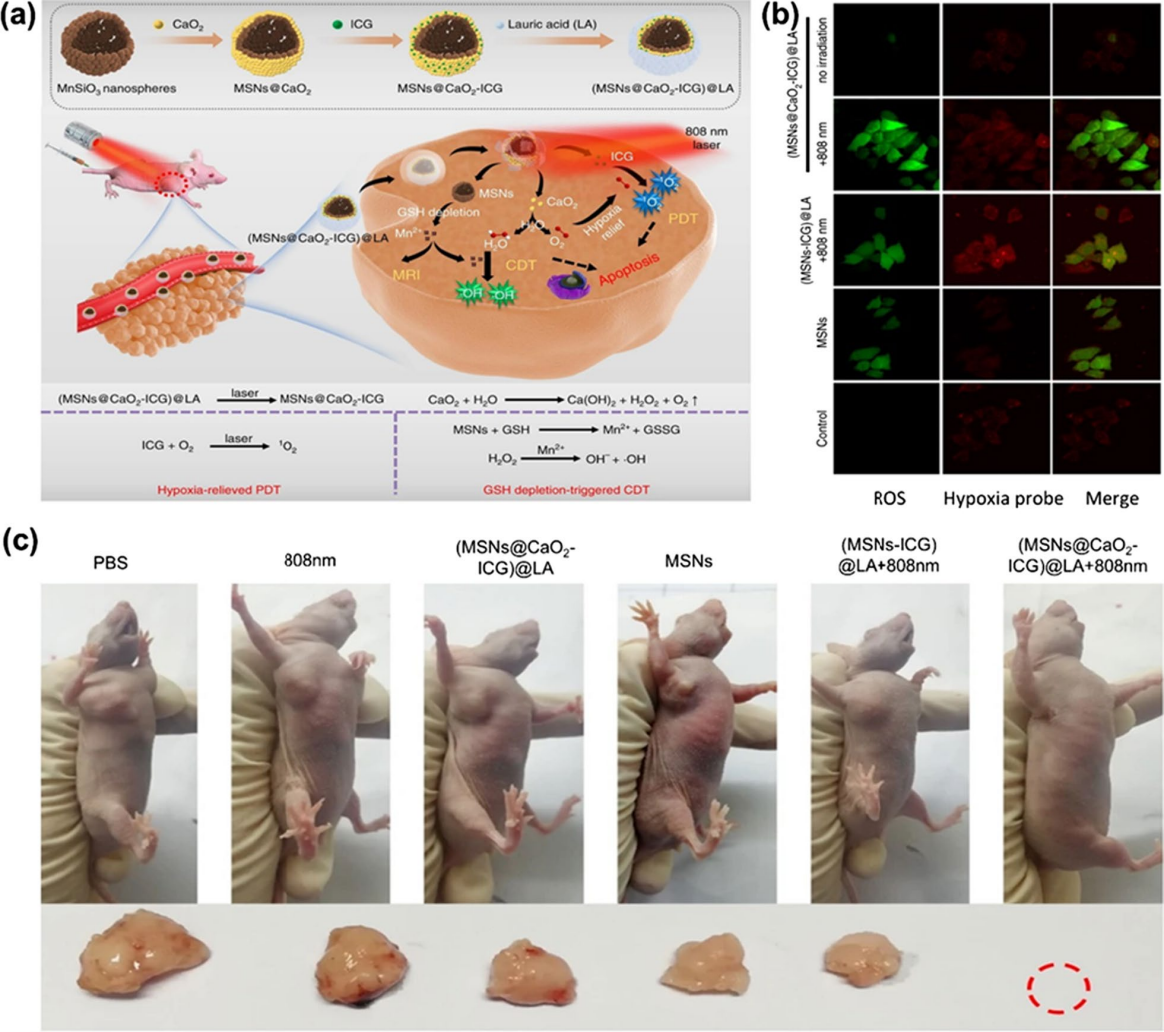
**a** The synthesis of (MSNs@CaO_2_-ICG)@LA and its action mechanism in cancer cells [136]. **b** Fluorescence images show ROS and hypoxia level in MCF-7 cells with different treatment under normoxia condition [136]. **c** Representative photos with different treatments of the mice and corresponding tumor tissues collected from different groups at 14 days. Reproduced with permission [136]. Copyright 2020, The Author(s)

Liu and co-workers [135] also constructed a nanoplatform((MSNs@CaO_2_-ICG) @LA) that could solve the poor therapeutic effect caused by high GSH content and low concentration of H_2_O_2_ in the TME. As shown in Fig. 15a, (MSNs@CaO_2_-ICG) @LA was prepared by loading CaO_2_ and indocyanine green (ICG) on manganese silicate (MSNs) and coating with the phase change material lauric acid (LA). This nanocatalyst can respond to the TME. MSNs was able to react with GSH and release Mn^2+^ Fenton-like ions. CaO_2_ was able to react with water under an acidic environment and generate much H_2_O_2_ and O_2_. Finally, there would be a large amount of ROS in the TME under the effect of Mn^2+^. More interestingly, these results were achieved by the introduction of the phase change material LA which would crack under the irradiation of 808 nm laser. In addition, photosensitizer ICG could synergistically enhance the concentration of ROS in cancer cells. O_2_ could react with it to produce ^1^O_2_ under laser irradiation. The in vivo and in vitro studies showed that (MSNs@CaO_2_-ICG)@LA could significantly improve the therapeutic effect, indicating that the combination of PDT and CDT has an excellent anticancer effect, as shown in Fig. 15b, c.

### Applications of F-NCs in SDT

Light is the external energy of PTT and PDT, which can stimulate material to interact with TME to kill cancer cells. But light as external energy has a fatal disadvantage. The maximum penetration depth of light below the skin tissue is 10 mm, which seriously limits the clinical applications of PDT and PTT. However, the penetration depth of ultrasound below the skin tissue is 10 cm, which can solve the problem of tumor treatment deep in the body. Compared with PTT and PDT, the principle of SDT is more complex. At present, there are many studies on the mechanism of SDT, but there is still no definite conclusion. Most researchers support the synergy of multiple mechanisms leading to cell death. Among them, ultrasonic cavitation, ROS, and ultrasonic induced apoptosis are the recognized reasons for cell death caused by SDT [136].

Similar to PDT, SDT is also a relatively mild treatment for cancer. However, the large size, low sensitivity, and insufficient osmotic capacity of the sonosensitizer at present result in the inadequate short-term treatment effect of SDT [137, 138]. In order to avoid the relatively long treatment cycle of SDT, it is necessary to improve the sonosensitizer. Some studies have proved that under the action of ultrasonic cavitation, the collapse of cavitation bubbles could cause the local liquid to occur violent turbulence, which could enhance the mass transfer rate of homogeneous or heterogeneous Fenton reagent system and consequently promote the therapeutic effect of CDT [139]. Therefore, the combination of CDT and SDT provides a new model to improve the effectiveness of cancer treatment.

**Fig. 16:**
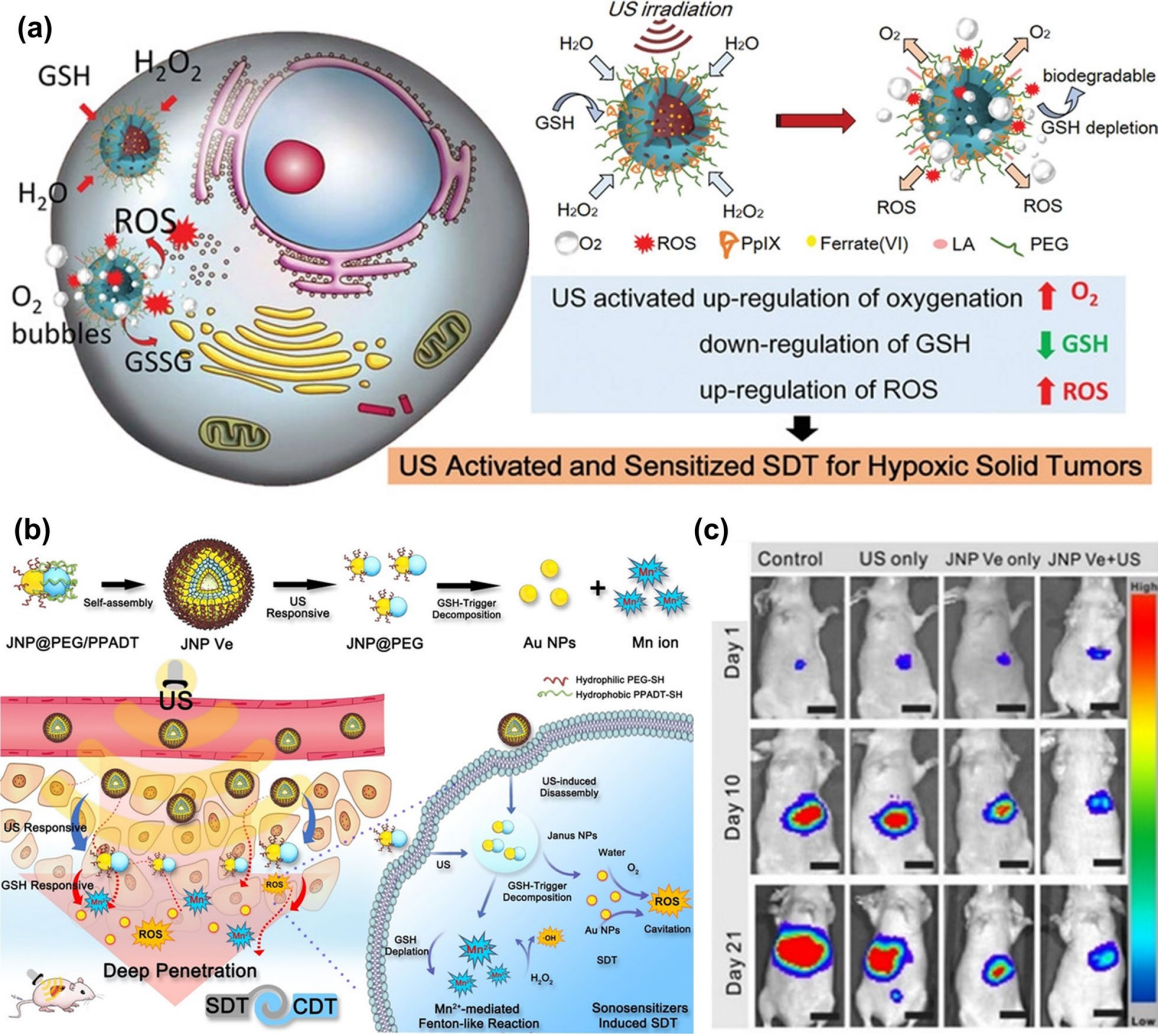
**a** Mechanism of Fe(VI)@HMONs in tumor cells. Reproduced with permission. [85] Copyright 2019, WILEY–VCH. **b** The preparation process of JNP@PEG/PPADT vesicles, and their osmosis and therapeutic mechanisms in tumors. [144]. **e** Representative images of mice at day 1, 10, and 21 after different treatments. Reproduced with permission [144]. Copyright 2020, Wiley–VCH

Sonosensitizers are generally divided into organic and inorganic sonosensitizers. Organic sonosensitizers generally include porphyrin and its derivatives, DOX, curcumin, etc. [136]*.* Organic sonosensitizers generally come from photosensitizers, and organic sonosensitizers have a short cycle in the body and are specifically recognized and discharged out of the body by the immune system [140]. Therefore, organic sonosensitizers are generally loaded in carriers with good stability, so as to avoid premature exposure of organic sonosensitizers and to be transported to the tumor site with minimum loss. For example, Huang et al. [141] carried organic sonosensitizer protoporphyrin (PpIX) onto HMONs and constructed a sonosensitizer MnPpIX@HMONs with Fenton effect via the coordination between Mn^2+^ and protoporphyrin. The therapeutic effect of MnPpIX@HMONs under the action of ultrasound was obviously better than that of single-use MnPpIX@HMONs, which demonstrates that the F-NCs can improve the effect of SDT. Fu et al. [85] also used HMONs to load protoporphyrin and at the same time adsorb FeO_4_^2−^ in the mesopore of HMONs by electrostatic adsorption. They used the phase change material lauric acid to coat Fe(VI)@HMONs-PpIX. A slight thermal effect induced by ultrasound could trigger the phase change of lauric acid, resulting in the release of FeO_4_^2−^. Subsequently, a series of oxidation reactions occurred in TME. FeO_4_^2−^ could reduce the concentration of GSH, and the Fenton reaction between Fe^2+^ and H_2_O_2_ could increase the ROS level in TME, as shown in Fig. 16a.

Inorganic sonosensitizers are more stable than organic sonosensitizers, and the most representative inorganic sonosensitizer is TiO_2_. In addition, inorganic sonosensitizers have less toxic and side effects on the human body, and it is easy to control the size and morphology of inorganic sonosensitizers so that inorganic sonosensitizers can accumulate in tumor cells better [142]. At present, the trend of designing inorganic sonosensitizers is to make inorganic sonosensitizers multifunctional, so as to realize multi-mode therapy. For example, sonosensitizer TiO_1+x_ and MnWO_x_ [50, 72] with anoxic structure could greatly improve the therapeutic effect of SDT since the Fenton-like Mn^2+^ and Ti^3+^ could catalyze the decomposition of H_2_O_2_. Moreover, the anoxic structure acts as a charge trap to limit the recombination of electron–hole pairs, thus improving the quantum yield of ROS. Recently, it has been demonstrated that ultra-small nanoscale Au NPs can also be used as an effective sonosensitizer in SDT. Lin et al. [143] prepared Janus Au-MnO NPs (JNPs) with an asymmetric particle size of 10 nm by the heteroepitaxial growth of MnO on one side of Au NPs. Then the hydrophobic ROS sensitive polymer poly-(1,4-phenyleneacetone dimethylene thioketal) (PPADT-SH) and hydrophilic PEG-SH were grafted onto the surface of Au by covalent bond Au–S. Eventually, the amphiphilic JNP@PEG/PPADT vesicles (JNP Ve) were prepared by oil in water emulsification (Fig. 16b). In cancer cells, the ROS-sensitive polymer PPADT cleaves under the action of ultrasound. MnO is exposed to TME and reacts with GSH to produce Mn^2+^ so that a large number of Au NPs exist alone in TME. The nano-size (5 nm) of Au NPs increases the possibility of ultrasonic cavitation to improve the level of ROS in TME and finally achieve the combined treatment of CDT and SDT, as shown in Fig. 16b. The in vivo experiments showed that the therapeutic effect of JNP Ve was greatly reduced without ultrasound (Fig. 16c), because PPADT could not be decomposed, resulting in a poor penetration ability of JNP Ve.

## Conclusions and outlook

**Fig. 17:**
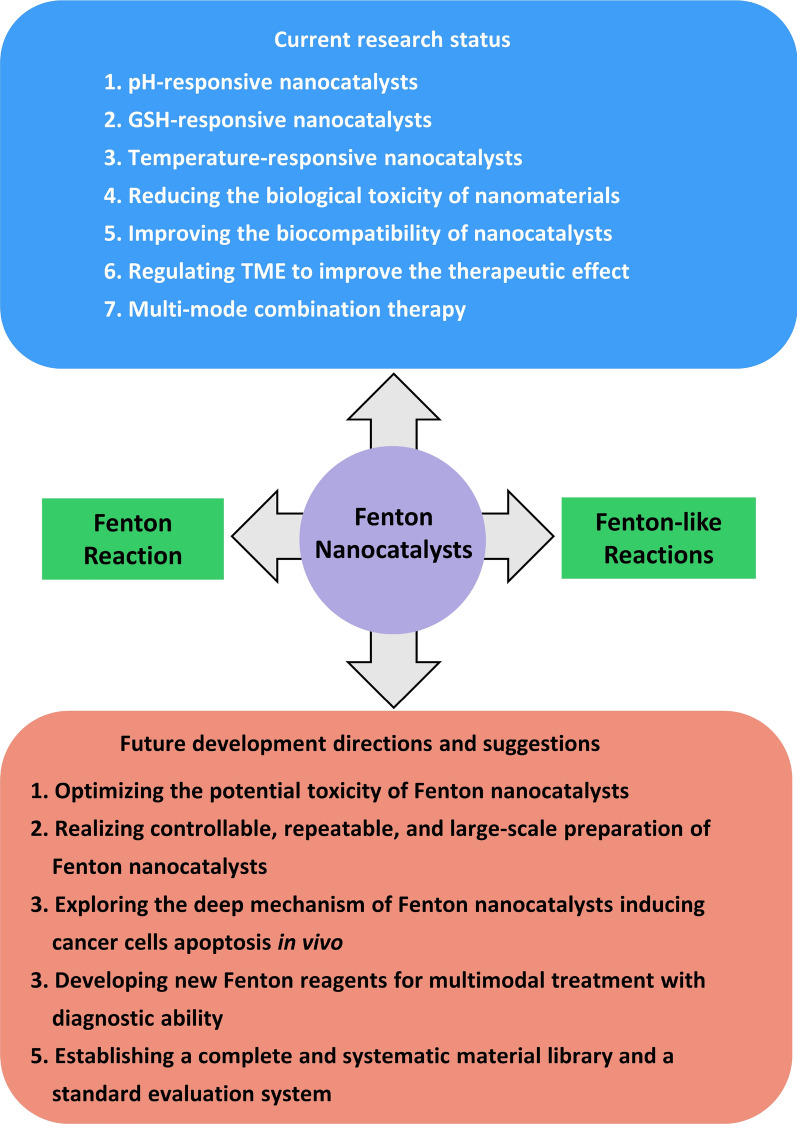
Research status and future directions of Fenton nanocatalysts in cancer treatment

This review has summarized the development of F-NCs in recent years by introducing their preparation process, action mechanism, and applications in cancer treatment. With regards to the deficiency of F-NCs, some effective strategies to improve their therapeutic effect are proposed. In addition, the development prospect of F-NCs in this emerging field has prospected, as shown in Fig. 17. The F-NCs are usually responsive to the TME which can be regulated to enhance the lethality of the nanoplatforms to cancer cells, showing huge potentials for anticancer treatments and significantly accelerating the pace of clinical anticancer of nanomaterials. Although F-NCs have made important advances in cancer therapy, some crucial issues that can promote F-NCs to enter the clinic must be considered.

First, reducing the potential toxicity of F-NCs. Metal-based F-NCs may have systemic toxicity problems (such as nervous system abnormalities, loss of organ metabolic function, etc.). The low biocompatibility of metal-based F-NCs and the low uptake of F-NCs by cancer cells are the key reasons for their systemic toxicity. Generally, there are two ways to improve the biocompatibility of F-NCs. One is to select natural and non-toxic biomaterials with good water solubility in the synthesis process of F-NCs; the other one is to improve its biocompatibility through surface engineering. The surface engineering of Fenton nanocatalyst mainly includes: (a) introducing polymers (such as polyethylene and polyhydroxy ethyl methacrylate) on the material surface to prevent the adsorption of specific proteins; (b) introducing bioactive substances (such as specific growth factors) on the surface to biomimetic the cell membrane to achieve the purpose of biocompatibility so as to realize the "invisibility" of F-NCs to normal cells and proteins. At present, the accumulation of F-NCs in tumors is mainly achieved by the EPR effect. However, the uptake rate of F-NCs by tumor cells is less than 10% (intravenous injection), which will increase the possibility of F-NCs entering human functional organs through blood circulation. Strengthening the uptake of tumor cells can be achieved by adjusting the size and shape of nanoparticles. Another effective method is to utilize tumor-targeting technology to improve the uptake of F-NCs and reduce the distribution of F-NCs in the body.

Second, realizing controllable, repeatable, and large-scale preparation of F-NCs. Due to the complex preparation process of F-NCs, it is difficult to synthesize the same nanomaterials quickly, accurately, and repeatedly. Therefore, some novel and simple preparation technics should be developed in future research, such as the microfluidic method, which can synthesize nanoparticles with uniform size, adjustable physical and chemical properties, and good repeatability through high-speed self-assembly. In addition, the synthesis cost of most current F-NCs is too high for large-scale production, so future research should focus on developing low-cost F-NCs.

Third, exploring the in-depth mechanism of F-NCs inducing cancer cell apoptosis in vivo. Most researchers believe that the apoptosis of cancer cells is caused by the oxidation of ROS, and this theory has been confirmed by in vitro experiments [144]. However, the TME is complex; this theory may not be suitable for the study of the interaction between F-NCs and cancer cells (inducing apoptosis) *in vivo*, which should be got more attention in future research. Exploring the specific mechanism of F-NCs in vivo is conducive to the design and development of multifunctional F-NCs.

Fourth, developing new Fenton reagents for multimodal treatment with diagnostic ability. Although many strategies have been proposed to improve CDT performance while the therapeutic effect is not very satisfactory. To completely eliminate malignancies, designing multifunctional F-NCs that can be used in other therapies is necessary. A large number of studies have proved that CDT combined with other treatments (such as PTT, PDT, SDT, immunotherapy, GT, etc.) can achieve the effect of “1 + 1 > 2” [145]. Particularly, F-NCs with the ability of diagnosis and multimodal therapy are more promising to enter the clinical stage. Because these nanomedicines can accurately diagnose the disease in real-time and treat it simultaneously. More importantly, we can monitor the curative effect and adjust the administration plan at any time in the whole treatment process, which is conducive to achieving the best treatment effect. Fortunately, some multifunctional F-NCs with imaging modes (such as magnetic resonance [146], ultrasonic imaging [147], photothermal imaging [148], and surface-enhanced Raman [149]) have been developed, which lays a solid foundation to realize the precise theranostics of cancer.

Fifth, a complete and systematic material library and a standard evaluation system should be established. The way of preparation, collection, storage, and the structure and morphology of the samples have a direct impact on the catalytic and therapeutic effect of the F-NCs, and there is no unified standard to evaluate the catalytic capacity and therapeutic efficacy of different F-NCs. The establishment of material library and evaluation system can direct the design of F-NCs with desirable properties.

In summary, F-NCs have broad application prospects in the field of anticancer therapy. We hope that through our continuous efforts, we can design NCs with high catalytic efficiency, excellent safety, and perfect performance to realize their clinical applications as soon as possible and bring the gospel to cancer patients in the near future.

## Supplementary Information

**Additional file 1: Table S1**. Typical Fe-based NCs with Fenton effect. **Table S2**. Representative Mn-based F-NCs with Fenton-like effect. **Table S3**. Other F-NCs with Fenton-like effect

## Data Availability

Not applicable.

## Abbreviations

CDT: Chemodynamic therapy
F-NCs: Fenton nanocatalysts
NCs: Nanocatalysts
NPs: Nanoparticles
TME: Tumor microenvironment
GSH: Glutathione
H_2_O_2_: Hydrogen peroxide
IARC: International Agency for Research on Cancer
ROS: Reactive oxygen species
^1^O_2_: Singlet oxygen
O_2_^2^^−^: Peroxide ion
O^2^^−^: Superoxide ion
•OH: Hydroxyl radical
PTT: Photothermal therapy
PDT: Photodynamic therapy
US: Ultrasound
IONPs: Superparamagnetic iron oxide nanoparticles
MRI: Magnetic resonance imaging
CDDP: Cis-platinum
LF: Lactoferrin
RGD_2_: RGD dimer
GOD: Glucose oxidase
MOFs: Metal–organic frameworks
EPR: Enhancing permeability and retention effect
DCA: Dichloroacetic acid
Fe(0): Zero-valent iron
PHNPs: Porous hollow Fe_3_O_4_ nanoparticals
PYSNPs: Porous yolk-shell Fe/Fe_3_O_4_ nanoparticals
T1-MRI: T1-weighted magnetic resonance imaging
LMN: Lm@MnO_2_
CA: Cinnamaldehyde
CLMNF: CA &LM@MnO_2_-HA nanoflowers
CNs: Carbon nanospheres
OAm: Oil amine
ODE: 1-Octadecene
MTT: Microwave thermal therapy
Mn-ZrMOF NCs: Mn-doped zirconium metal–organic framework nonocubes
HIFU: High-intensity focused ultrasound
MD@Lip: DCA-modified octahedral metal–organic framework MOF-Fe^2^^+^
TAM: Tamoxifen
AMP: Adenosine monophosphate
ATP: Adenosine triphosphate
AMPK: AMP-activated protein kinase
FcPWNP: Cysteine-iron phosphotungstate chelate nanoparticle
ApDC: Apder-prodrug conjugate
T: Tetraoxane
PLGA: P LGA
CAT: Catalase
DOX: Doxorubicin
CA: Cinnamaldehyde
Vk3: Vitamin k3
Lap: β-Lapachone
NQO1: NAD(P)H quinone oxidoreductase-1
CP: Copper peroxide nanopoints
DHA: Dihydroartemisinin
HMONs: Hollow mesoporous silica
BTDS: 4,4,13,13-Tetraethoxy-3,14-dioxa-8,9-dichiath-4,13-disilahxadecane
S–S: Disulfide bond
PTAs: Photothermal agents
DNA-UCNP-Au: DNA-mediated upconversion and gold nanoparticles hybrid hydrogels
ASPA: Activatable semiconducting polymeric nanoantagonist
AuNCs@SiO_2_: Silica-encapsulated self-assembled gold nanochains
FePS_3_ NSs: FePS_3_ nanosheets
NIR-I: Near Infrared-I
NIR-II: Near Infrared-II
2D: Two-dimension
BPNS: Black phosphorus nanosheets
LDNPs: Lanthanide-doped nanoparticles
CMSN: Copper/manganese silicate nanospheres
UC: Upconversion
DC: Downconversion
ICG: Indocyanine green
MSNs: Manganese silicate
LA: Lauric acid
PpIX: Protoporphyrin
JNPs: Janus Au-MnO nanoparticles
PPADT-SH: Poly-(1,4-phenyleneacetone dimethylene thioketal)
JNP Ve: JNP@PEG/PPADT vesicles
