# Supplementary material for: Tumor microenvironment-responsive fenton nanocatalysts for intensified anticancer treatment
Source: J Nanobiotechnology. 2022 Feb 5;20:69. doi: 10.1186/s12951-022-01278-z (PMC8817594; doi:10.1186/s12951-022-01278-z)
Supplement: Supplementary file 1 — Additional file 1: Table S1. Typical Fe-based NCs with Fenton effect. Table S2. Representative Mn-based F-NCs with Fenton-like effect. Table S3. Other F-NCs with Fenton-like effect [file 12951_2022_1278_MOESM1_ESM.docx]

| Num | Fe-Fenton nanocatalysts | Therapeutic modality | Functional mechanism | Ref |
| --- | --- | --- | --- | --- |
| 1 | MoS_2_-ALG-Fe/GOD | PTT/Starvation therapy (ST)/CDT | GOD can oxidize glucose to produce H_2_O_2_. Fe^3+^ can react with MoS_2_ to generate Fe^2+^ and MoO_4_^2-^. Fe^2+^ ions induce Fenton reaction, MoS_2_ has high photothermal conversion ability for PTT. | [1] |
| 2 | Cu_5_FeS_4_ | PTT/CDT | With the increase of the Cu/Fe ratio, the photothermal conversion ability is enhanced, and Cu_5_FeS_4_ has the best performance and shows the temperature-responsive Fenton catalytic performance. | [2] |
| 3 | PEG-Fns | PDT/CDT | Under blue light, Ferrihydrite nanoparticles can release Fe^2+^ and promote ROS/Fe^2+^-related DNA fragmentation and glutathione peroxide 4 (GPx4) inhibition. This approach can induce the polarization of tumor-associated macrophages from tumor-promoting M2-type to the tumor-killing M1-type. | [3] |
| 4 | LET-6 | CDT+PTT | LET-6 is composed of tPy-Cy-Fe and DSPE-PEG. tPy-Cy serves as fluorescence (FL) and photoacoustic (PA) dual-modality imaging contrast agent, photothermal transducer as well as iron chelator. Under 808nm laser irradiation, the structure of LET-6 will be cracked, and Fe^2+^ is released to enhance PTT. | [4] |
| 5 | FeWO_x_-PEG | CDT | FeWO_x_ contains polyvalent metal elements (Fe^2+^/^3+^, W^5+^/^6+^), which is able to achieve the efficient catalytic decomposition of H_2_O_2_. Fe^3+^/W^6+^ are reduced by endogenous GSH to produce low-state metal ions, and a cascade of biological reactions are realized. | [5] |
| 6 | Fe-TiO_2_ NDs | SDT/CDT | By doping Fe with TiO_2_, the bandgap of TiO_2_ can be reduced so that Fe-TiO_2_ has a better acoustic dynamics effect than pure TiO_2_. In addition, Fe can also trigger the Fenton reaction so that CDT and SDT can be used together to kill 4T1 cells | [6] |
| 7 | Hollow Fe_3_O_4_ mesocrystals (MCs) | Magnetic hyperthermia (MH)/CDT | Fe_3_O_4_ MCs have an excellent magneto-thermal conversion efficiency. Compared with Fe_3_O_4_ PC_S_, Fe_3_O_4_ MCs have a higher ratio of Fe^2+^/Fe^3+^ and more oxygen defects, so it shows a higher peroxidase activity, which can realize the combination therapy of MH and CDT, and induce apoptosis of 4T1 cells. | [7] |
| 8 | SnFe_2_O_4_ | Heterogeneous Fenton reactions | SnFe_2_O_4_ nanocrystals is a heterogeneous Fenton nanocatalyst used in the selective killing of lung cancer. The internalized SnFe_2_O_4_ nanocrystals can convert endogenous H_2_O_2_ into ROS. | [8] |

**Table 1** Typical Fe-based NCs with Fenton effect.

**Table 2.** Representative Mn-based F-NCs with Fenton-like effect.

| Num | Mn-based Fenton-like NCs | Therapeutic modality | Functional mechanism | Ref |
| --- | --- | --- | --- | --- |
| 1 | MS@MnO_2_ | CDT | MnO_2_ undergo a redox reaction with GSH and produced Mn^2+^, which can catalyze the decomposition of H_2_O_2_ to produce ROS and finally cause apoptosis of cancer cells. | [9] |
| 2 | HMCMD | PTT/CDT | The yolk-shell CuS has a good photothermal conversion efficiency. MnO_2_ can oxidize GSH to produce Mn^2+^ with Fenton effect. The combination therapy of CDT and PTT can effectively kill MCF-7 cells. | [10] |
| 3 | MCMSNs | PDT/CDT | Porous Cu/Mn silicon nanospheres (MCMSNs) were coated by cancer cell membrane. When MCMSNs enter cancer cells, they can react with GSH to produce Cu^+^ and Mn^2+^, triggering the Fenton-like reaction. | [11] |
| 4 | PLTM-HMnO_2_ | CT/CDT | The platelet membrane enhances the biocompatibility of the nanoplatforms, and MnO_2_ reacts with GSH to release Mn^2+^, catalyzing the Fenton reaction. | [12] |
| 5 | Mn-DNA | CDT | Mn-DNA coordination NPs can sensitively release Mn^2+^ in tumors, which can consum endogenous H_2_O_2_ through Fenton-like reactions to produce highly toxic ROS. | [13] |
| 6 | Mn-Cu bimetallic complex | CDT | Mn-Cu bimetallic complex can release Mn^2+^ in tumor tissues, which can improve the level of ROS. The Cu complex can react with GSH，thereby enhancing the efficiency of CDT. | [14] |
| 7 | FeMn-LDH | PTT/PDT/CDT | FeMn-LDH was loaded on UCSPs, which was covalently coated by mesoporous silica and Ce6. UCSPs endow FeMn-LDH with photothermal conversion capability. In acidic environment, MnFe-LDH is decomposed to generate Fe^3+^ and Mn^2+^ Fenton ions to produce ROS, enhancing UCSP-guided PTT. | [15] |
| 8 | MNS-GOD | ST/PTT/CDT | Manganese dioxide nanosheets with a large specific surface area can load glucose oxidase, MNS/GOD has good photothermal conversion ability, can carry out glucose oxidation and hydrogen peroxide decomposition cycle reaction and realize ST/PTT/CDT combined treatment. | [16] |

**Table 3.** Other F-NCs with Fenton-like effect

| Num | Other Fenton-like NCs | Therapeutic modality | Functional mechanism | Ref |
| --- | --- | --- | --- | --- |
| 1 | CuO@CNs-DOX | PTT/CDT | CuO can respond to TME to produce Cu^2+^, which can catalyze the decomposition of H_2_O_2_, resulting in Fenton-like reaction. | [17] |
| 2 | AgNPs-TAMRA-DNA@GO | CDT | Ag NPs can catalyze H_2_O_2_ decomposition to generate hydroxyl radical in an acidic environment, resulting in Fenton-like reaction. | [18] |
| 3 | AgNPs | CDT | Ag NPs can catalyze H_2_O_2_ to generate •OH and Ag^+^ ions in acidic environment, resulting in Fenton-like reaction. | [19] |
| 4 | V-TiO_2_-PEG | CDT/SDT | This platform can regulate the TME. V^5+^ ions have the ability to catalyze the decomposition of H_2_O_2_ and can oxidize GSH. | [20] |
| 5 | Ru@SiO_2_-PA | CDT | Ru can produce Ru^2+^ in solid tumors with an acidic environment and catalyze the decomposition of H_2_O_2_. | [21] |
| 6 | Co^2+^-HCO^3-^ | CDT | The ability of Co^2+^ to catalyze the decomposition of H_2_O_2_ is not strong, but the Fenton-like effect will be greatly enhanced after the reaction of the complex with HCO^3-^ ions, which can effectively degrade organic pollutants. | [22] |
| 7 | g-C_3_N_4_ | **—** | The Fenton-like activity of g-C_3_N_4_ is mainly related to the -OH functional group on the structure of g-C_3_N_4_, oxygenated functional groups (hydroquinone-like groups) can reduce the H_2_O_2_ and generate •OH. | [23] |
| 8 | GaIn@Pt NPs | PTT/CDT | Through *in-situ* redox reaction, Pt is assembled on the surface of GaIn alloy. The installation of Pt improves the photothermal conversion efficiency and thermal stability of GaIn. More importantly, Pt can convert H_2_O_2_ to ROS for killing tumor cells. | [24] |

**Abbreviations**

| MoS_2_-ALG-Fe/GOD | MoS_2_ and GOD-containing sodium alginate (ALG)-Fe^3+^ (MAF) hydrogel |
| --- | --- |
| PEG-Fns | PEG-coated ferrihydrite nanoparticles |
| ST | Starvation therapy |
| GP_X_4 | Glutathione peroxide4 |
| tPy-Cy-Fe | Fe^2+^ chelated 4′-(amino-methyl phenyl)-2,2′:6′,2′′-terpyridine modified cyanine |
| FL | Fluorescence |
| DSPE-PEG | Distearoylphosphoethanolamine-polyethylene glycol |
| PA | Photoacoustic |
| MCs | Mesocrystals |
| MH | Magnetic hyperthermia |
| MS@MnO_2_ | MnO_2_-coated mesoporous silica nanoparticles |
| MCMSNs | Porous Cu/Mn silicon nanospheres |
| PLTM | Platelet membrane |
| HMnO_2_ | Hollow MnO_2_ nanoparticles |
| FeMn-LDH | Fe-Mn layered double hydroxides |
| MNS-GOD | GOD armed manganese dioxide nanosheets |
| CuO@CNs | CuO-decorated carbon nanoplatforms |
| Ru@SiO_2_-PA | Ruthenium-loaded palmitoyl ascorbate (PA)-modified mesoporous silica |

**Reference**

1. Zhou LL, Zhao JL, Chen YK, Zheng YT, Li JF, Zhao JY, et al. MoS_2_-ALG-Fe/GOx hydrogel with Fenton catalytic activity for combined cancer photothermal, starvation, and chemodynamic therapy. Colloid Surface B. 2020;195.

2. Wang ZJ, Wang Y, Guo HH, Yu N, Ren Q, Jiang Q, et al. Synthesis of one-for-all type Cu_5_FeS_4_ nanocrystals with improved near infrared photothermal and Fenton effects for simultaneous imaging and therapy of tumor. Journal of Colloid and Interface Science. 2021;592:116-126.

3. Yang YC, Tian Q, Wu S Q, Li YX, Yang K, Yan Y, et al. Blue light-triggered Fe^2+^-release from monodispersed ferrihydrite nanoparticles for cancer iron therapy. Biomaterials. 2021;271:120739.

4. He T, Yuan Y, Jiang C, Blum N T, He J, Huang P, et al. Light‐Triggered Transformable Ferrous Ion Delivery System for Photothermal Primed Chemodynamic Therapy. Angewandte Chemie International Edition. 2021;60:6047-6054.

5. Gong F, Chen MC, Yang NL, Dong ZL, Tian LL, Hao Y, et al. Bimetallic Oxide FeWO_X_ Nanosheets as Multifunctional Cascade Bioreactors for Tumor Microenvironment Modulation and Enhanced Multimodal Cancer Therapy. Adv. Funct. Mater. 2020;30:202753.

6. Bai S, Yang N, Wang X, Gong F, Dong Z, Gong Y, et al. Ultrasmall Iron-Doped Titanium Oxide Nanodots for Enhanced Sonodynamic and Chemodynamic Cancer Therapy. ACS Nano. 2020;14:15119-15130.

7. Du WX, Liu TZ, Xue FF, Cai XJ, Chen A, Zheng YY, et al. Fe_3_O_4_ Mesocrystals with Distinctive Magnetothermal and Nanoenzyme Activity Enabling Self-Reinforcing Synergistic Cancer Therapy. Acs Appl Mater Inter. 2020;12:19285-19294.

8. Lee KT, Lu YJ, Mi FL, Burnouf T, Wei YT, Chiu SC, et al. Catalase-Modulated Heterogeneous Fenton Reaction for Selective Cancer Cell Eradication: SnFe_2_O_4_ Nanocrystals as an Effective Reagent for Treating Lung Cancer Cells. Acs Appl Mater Inter. 2017;9:1273-1279.

9. Lin LS, Song JB, Song L, Ke KM, Liu YJ, Zhou ZJ, et al. Simultaneous Fenton-like Ion Delivery and Glutathione Depletion by MnO2-Based Nanoagent to Enhance Chemodynamic Therapy. Angew Chem Int Edit. 2018;57:4902-4906.

10. Gu D, An P, He X, Wu H, Gao Z, Li Y, et al. A novel versatile yolk-shell nanosystem based on NIR-elevated drug release and GSH depletion-enhanced Fenton-like reaction for synergistic cancer therapy. Colloids Surf B Biointerfaces. 2020;189:110810.

11. Liu C, Wang D, Zhang S, Cheng Y, Yang F, Xing Y, et al. Biodegradable Biomimic Copper/Manganese Silicate Nanospheres for Chemodynamic/Photodynamic Synergistic Therapy with Simultaneous Glutathione Depletion and Hypoxia Relief. ACS Nano. 2019;13:4267-4277.

12. Wang H J, Bremner DH, Wu K H, Gong XR, Fan Q, Xie XT, et al. Platelet membrane biomimetic bufalin-loaded hollow MnO_2_ nanoparticles for MRI-guided chemo-chemodynamic combined therapy of cancer. Chemical Engineering Journal. 2020;382:122848.

13. Lin L, Yu J, Lu H, Wei Z, Chao Z, Wang Z, et al. Mn-DNA coordination of nanoparticles for efficient chemodynamic therapy. Chem Commun (Camb). 2021;57:1734-1737.

14. Cao S, Fan J, Sun W, Li F, Li K, Tai X, et al. A novel Mn-Cu bimetallic complex for enhanced chemodynamic therapy with simultaneous glutathione depletion. Chem Commun (Camb). 2019;55:12956-12959.

15. Jia T, Wang Z, Sun Q, Dong S, Xu J, Zhang F, et al. Intelligent Fe-Mn Layered Double Hydroxides Nanosheets Anchored with Upconversion Nanoparticles for Oxygen-Elevated Synergetic Therapy and Bioimaging. Small. 2020;16:e2001343.

16. He T, Xu H, Zhang Y, Yi S, Cui R, Xing S, et al. Glucose Oxidase-Instructed Traceable Self-Oxygenation/Hyperthermia Dually Enhanced Cancer Starvation Therapy. Theranostics. 2020;10:1544-1554.

17. Jiang F, Ding B, Zhao Y, Liang S, Cheng Z, Xing B, et al. Biocompatible CuO-decorated carbon nanoplatforms for multiplexed imaging and enhanced antitumor efficacy via combined photothermal therapy/chemodynamic therapy/chemotherapy. Science China Materials. 2020;63:1818-1830.

18. Duan L Y, Wang YJ, Liu JW, Wang YM, Li N, Jiang JH. Tumor-selective catalytic nanosystem for activatable theranostics. Chem Commun (Camb). 2018;54:8214-8217.

19. He W, Zhou YT, Wamer WG, Boudreau MD, Yin JJ. Mechanisms of the pH dependent generation of hydroxyl radicals and oxygen induced by Ag nanoparticles. Biomaterials. 2012;33:7547-55.

20. Wang XW, Wang XY, Zhong XY, Li GQ, Yang ZJ, Gong YH, et al. V-TiO_2_ nanospindles with regulating tumor microenvironment performance for enhanced sonodynamic cancer therapy. Applied Physics Reviews. 2020;7:041411-1.

21. Sun D, Wang Z, Zhang P, Yin C, Wang J, Sun Y, et al. Ruthenium-loaded mesoporous silica as tumor microenvironment-response nano-fenton reactors for precise cancer therapy. J Nanobiotechnology. 2021;19:98.

22. Xu A, Li X, Ye S, Yin G, Zeng Q. Catalyzed oxidative degradation of methylene blue by in situ generated cobalt (II)-bicarbonate complexes with hydrogen peroxide. Applied Catalysis B: Environmental. 2011;102:37-43.

23. Oliveira WL, Ferreira MA, Mourao H, Pires MJM, Ferreira V, Gorgulho HF, et al. Heterogeneous Fenton-like surface properties of oxygenated graphitic carbon nitride. J Colloid Interface Sci. 2021;587:479-488.

24. Yang N, Gong F, Zhou Y, Hao Y, Dong Z, Lei H, et al. A general in-situ reduction method to prepare core-shell liquid-metal/metal nanoparticles for photothermally enhanced catalytic cancer therapy. Biomaterials. 2021;277:121125.
